# Advances of Electrochemical and Electrochemiluminescent Sensors Based on Covalent Organic Frameworks

**DOI:** 10.1007/s40820-023-01249-5

**Published:** 2023-11-30

**Authors:** Yue Cao, Ru Wu, Yan-Yan Gao, Yang Zhou, Jun-Jie Zhu

**Affiliations:** 1 Key Laboratory for Organic Electronics and Information Displays (KLOEID) and Institute of Advanced Materials (IAM), Nanjing University of Posts and Telecommunications (NJUPT), Nanjing, 210023 People’s Republic of China; 2 State Key Laboratory of Analytical Chemistry for Life Science, School of Chemistry and Chemical Engineering, Nanjing University, Nanjing, 210023 People’s Republic of China

**Keywords:** Covalent organic frameworks, Electrochemistry, Electrochemiluminescence, Sensors

## Abstract

Covalent organic frameworks (COFs), a rapidly developing category of crystalline conjugated organic polymers, possess highly ordered structures, large specific surface areas, stable chemical properties, and tunable pore microenvironments. Since the first report of boroxine/boronate ester-linked COFs in 2005, COFs have rapidly gained popularity, showing important application prospects in various fields, such as sensing, catalysis, separation, and energy storage. Among them, COFs-based electrochemical (EC) sensors with upgraded analytical performance are arousing extensive interest. In this review, therefore, we summarize the basic properties and the general synthesis methods of COFs used in the field of electroanalytical chemistry, with special emphasis on their usages in the fabrication of chemical sensors, ions sensors, immunosensors, and aptasensors. Notably, the emerged COFs in the electrochemiluminescence (ECL) realm are thoroughly covered along with their preliminary applications. Additionally, final conclusions on state-of-the-art COFs are provided in terms of EC and ECL sensors, as well as challenges and prospects for extending and improving the research and applications of COFs in electroanalytical chemistry.

## Highlights

- Covalent organic frameworks (COFs) show enormous potential for building high-performance electrochemical sensors due to their high porosity, large specific surface areas, stable rigid topology, ordered structures, and tunable pore microenvironments.
- The basic properties, monomers, and general synthesis methods of COFs in the electroanalytical chemistry field are introduced, with special emphasis on their usages in the fabrication of chemical sensors, ions sensors, immunosensors, and aptasensors.
- The emerged COFs in the electrochemiluminescence realm are thoroughly covered along with their preliminary applications.

## Graphical abstract

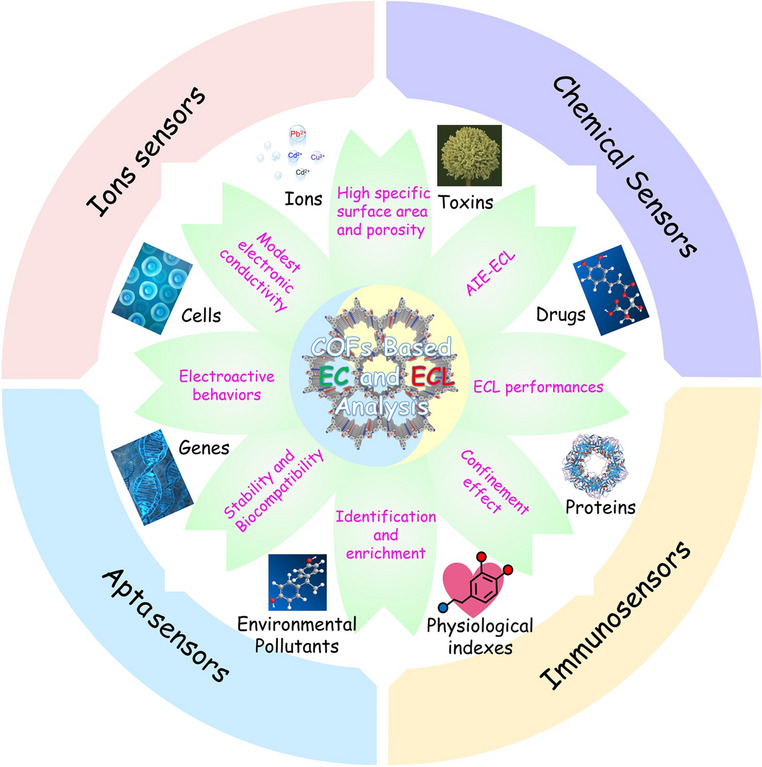

## Introduction

Since the discovery of boron-containing covalent organic frameworks (COFs) by Yaghi’s group in 2005 [1], this new class of porous crystalline materials has attracted widespread attention across several scientific communities [2–5]. Typically, COFs are formed via the polymerization of light elements, which are known to form robust covalent bonds in well-established and useful topologies [2, 3]. As a kind of porous polymer, crystalline COFs are characterized by a large specific surface area, high porosity, stable rigid topology, and tunable pore structure [4, 5]. Thus, with the rapid development of synthetic strategies and the properties disclosure of COFs in all aspects, a huge surge has emerged in their research and usage, especially in the fields of sensing, catalytic technology, optoelectronic devices, energy storage, pollutants treatment, etc. [6–11].

Electrochemistry has been recognized as a versatile analytical tool over the past few decades and remains competitive in the face of growing demands in life analysis, environmental monitoring, and food inspection [12, 13]. Known for superiorities in high sensitivity, low cost, fast response, simple equipment, and easy miniaturization, electrochemical (EC) sensors are devices that convert EC data between target analytes or sensing elements into analytical signals with application values [14–16]. Electrochemiluminescence (ECL), an important branch of EC technology, describes the light-emitting phenomenon initiated by the reactions of electro-generated radicals [17, 18]. During this process, the EC signals are converted into optical outputs, effectively avoiding EC background interference [19]. Since the luminescence source comes from the redox reactions on the electrode surface, ECL requires no external light source, displaying almost zero background [20]. Thus, ECL methodology, with characteristics of high sensitivity, outstanding controllability, and low-cost instruments, has aroused extensive research interest in clinical diagnosis, environmental monitoring, food analysis, etc. [17–20]. No matter the EC or ECL analysis, however, the essence is an EC reaction, that is, the electron-gain and loss reaction of electroactive species in the vicinity of the electrode [21]. Therefore, the established electrode surfaces afford the analytical performance of EC sensors, which is crucial for improving the key technical indicators such as sensitivity, stability, repeatability, and selectivity [17, 22].

It is known that EC sensors rely most on multifarious electroactive or catalytic materials on the electrodes, even though the EC detection methods are much the same [22–25]. Thus, developing and searching for high-quality electrode materials is particularly significant [26–28]. Microporous frameworks, such as inorganic zeolites, metal–organic frameworks (MOFs) [29–34], and organic COFs with high porosity and crystallinity are demonstrated to significantly enhance the analytical performance of EC sensors [35, 36]. Each one has its own superiorities and unique selling points [37–42], and their comparisons are summarized in Table 1 [43–45]. Thereinto, COFs as a brand-new category of porous crystalline materials have rapidly become a study focus in this area. The essence is that the super-micropores, high specific surface area, excellent stability, and modest conductivity of COFs guarantee sufficient active sites and favorable energy/mass transfer, while remarkable diversity and designability of the pore size, skeleton structure, and composition can regulate their EC behaviors.

**Table 1:** Comparisons of the microporous frameworks of zeolites, MOFs, and COFs

Materials	Micropore	Crystallinity	Stability	Conductivity	Diversity and designability	Solubility	Unique selling points	Research status
Zeolites	Ultra-micropores	Very high	Good	Poor	Good	No	Low cost; stability; commercialization	Active
MOFs	Ultra-micropores	Very high	Poor to good	Poor	Excellent	No	Precise control; ions centers	Hot
COFs	Super-micropores	Modest to high	Excellent	Modest	Excellent	No	Easy regulation; stability; organic	Very hot

**Fig. 1:**
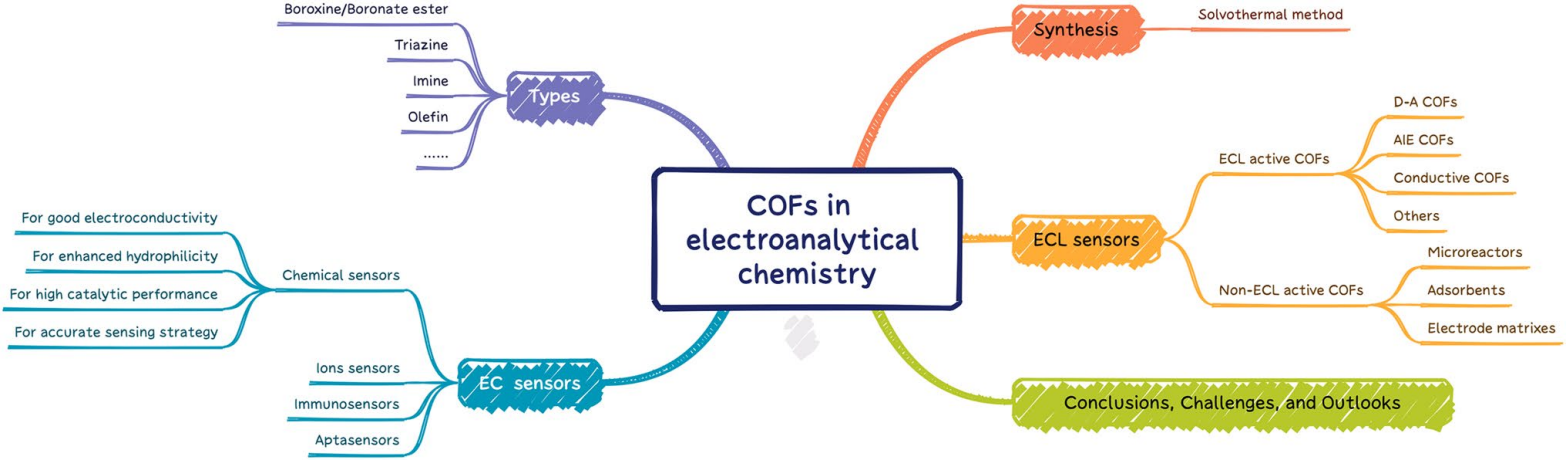
Flow chart representing the contents review article

Considering the great potential of the emerging COFs for building high-performance EC devices [45], this review primarily focuses on the corresponding analysis usages in electroanalytical chemistry (Fig. 1). To the best of our knowledge, there are only a few reviews partly regarding COFs in EC sensing [46–49], and no specific review that describes the role of COFs in the ECL domain to date. Hence, this review aims to make a comprehensive summary of the reports on COFs in electroanalytical chemistry in the last few years. We summarize the basic characteristics, monomers, and synthesis methods of COFs used in this field. Then, we introduce the EC analytical applications based on functional COFs systematically, including the fabrication of chemical sensors, ions sensors, immunosensors, and aptasensors. Also, COFs directly designed as ECL active emitters and non-ECL active matrixes, as well as their preliminary ECL sensing applications, are further highlighted. Finally, we discuss the challenges and future trends that COFs will face in the field of electroanalytical chemistry.

## Basic Characteristics and Synthetic Methods

Thanks to several impressive reviews on the structural features and synthesis of COFs [2, 3, 5], deep dives into this part will be attempted only regarding COFs in terms of electroanalytical chemistry in this review. Generally, COFs are created by the polymerization of monomers with symmetric reactive groups following specific geometries. During condensation, covalent bonds are spatially confined in either a two- or three-dimensional (2D or 3D) manner or geometrically directed to generate extended and ordered 2D or 3D crystal structures [50, 51]. Metal ions coordination or functional groups can be predesigned into the motifs or introduced through post-modification [6]. Moreover, the presence of heteroatoms within COFs creates special microenvironments for redox-active centers or specific target binding [52, 53]. As such, COFs will advance fundamental understanding and applications to shine in the field of EC analysis.

**Fig. 2:**
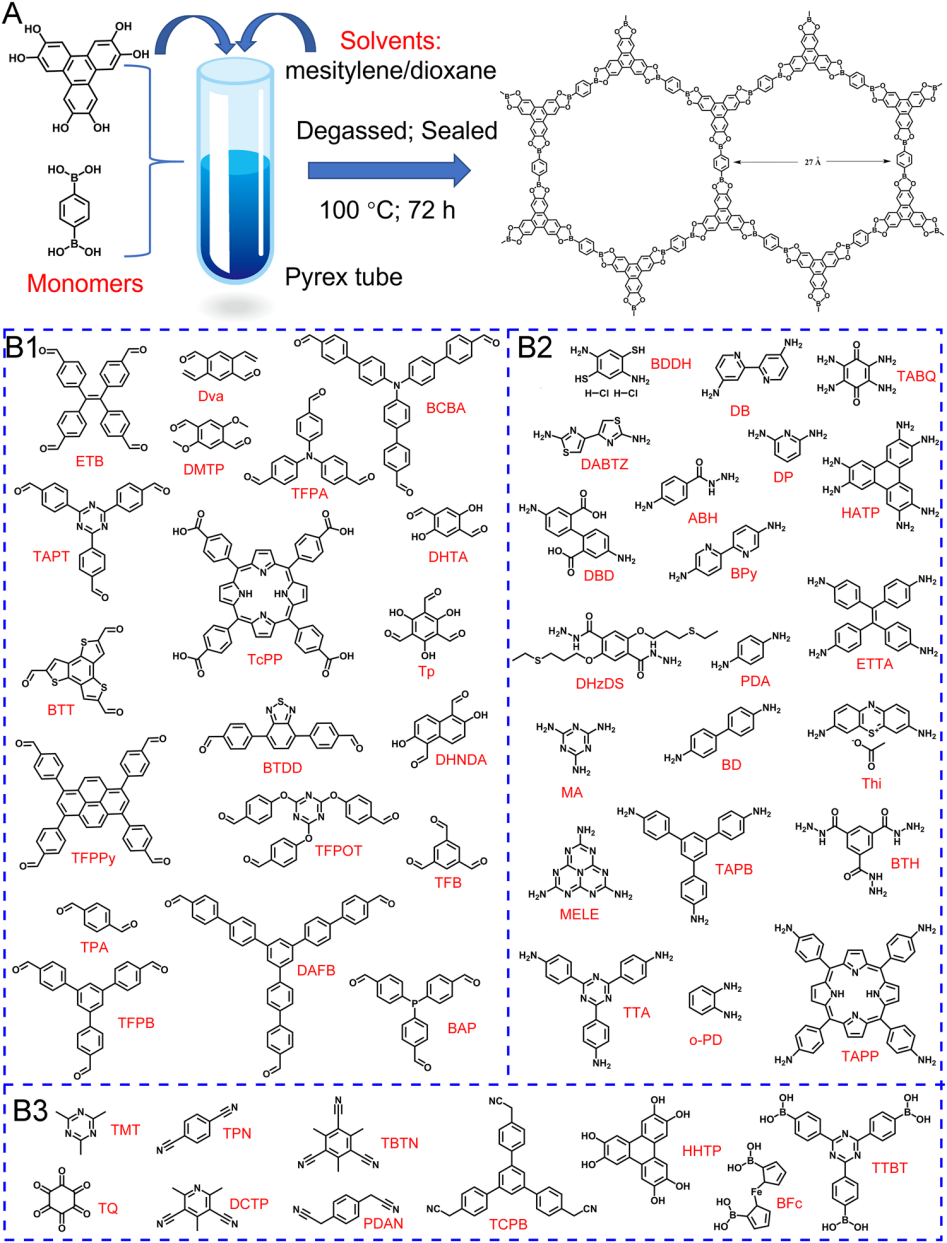
**A** The synthesis diagram of the boronate ester-linked COFs via a traditional solvothermal method; Summary of the **B1** aldehyde, **B2** amino, and **B3** other monomers for the synthesis of COFs reported in electroanalytical chemistry

Nowadays, various types of COFs are reported based on their dynamic covalent bonds, such as boronate ester, imine, olefin, *β*-ketoenamine, hydrazine, azine, and enamine [2–5, 54]. To the best of our knowledge, the formula of the monomers and their abbreviations used for the construction of COFs-based EC sensors are collected in Fig. 2B, mainly containing aldehyde, amino, cyano, triazine, hydroxyl, carbonyl, and boronic acid groups. The earliest reported boroxine/boronate ester-based COFs might be not stable enough to obtain available EC sensors in aqueous solutions or humid environments because of the electron-deficient nature of boron [1]. Triazine-containing COFs, also referred as covalent triazine skeletons, can be created by cyclic trimerization/hydroxyl aldol condensation reactions of nitrile. In comparison with boron-COFs, they have better stability and several EC usages, but they are commonly synthesized under harsh reaction conditions and with poor crystallinity [55, 56]. The imine-linked COFs are Schiff bases formed by condensation of amino and aldehyde groups, which have excellent crystallinity and structural regularity [57]. In particular, COFs with the imine linkage exhibit good chemical and thermal stability [4, 23]. For example, Xu et al. designed a porous imine-based COF with remarkable stability against water, strong bases, and strong acids by incorporating methoxy motifs into pore skeletons to enhance the interlayer interactions [4]. Until now, imine is still the most reported linkage in COFs preparation for EC sensing applications. Nevertheless, the electrical conductivity of these all-organic COFs is still limited due to the poor charge transfer ability of the linkages between the aromatic motifs [58]. The *sp*^2^ carbon-conjugated COFs with olefin linkages allow for acceptable electrical conductivity, beneficial to constructing EC sensors [59]. For the reader’s convenience, Table 2 summarizes the monomers and linkages of idiographic COFs in EC sensors.

**Table 2:** Summary of main basic characteristics of various COFs and their EC sensing applications

	COFs	Linkages	Monomers	Derived composites	EC methods	Analytes	Limit of detection	Refs.
Chemical sensors	COF_TAPB-TPA_	Imine	TAPB, TPA	COF/NH_2_-CNTs	DPV	Furazolidone	77.5 nM	[60]
	COF_BTH-Tp_	*β*-ketoenamine	BTH, Tp	COF/NH_2_-CNTs	DPV	Nitrofural	3.2 nM	[61]
	COF_TAPB-Dva_	Imine	TAPB, Dva	COF@graphitized MWCNTs	DPV	Diuron	0.08 μM	[62]
	COF_BD-TFPPy_	Imine	BD, TFPPy	COF/MWCNTs	DPV	Catechol, hydroquinone	0.36, 0.38 μM	[63]
	COF_TAPB-TFPB_	Imine	TAPB, TFPB	COF/Ox-MWCNTs	DPV	Dopamine, uric acid	0.073, 0.063 μM	[64]
	COF_ABH-TFPOT_	Imine	ABH, TFPOT	COF/polyaniline	DPV	Sulfamethoxazole	0.107 μM	[65]
	COF_TAPB-TPA_	Imine	TAPB, TPA	COF/AuNPs	SWV	Enrofloxacin	0.041 μM	[66]
	COF_TAPB-DMTP_	Imine	TAPB, DMTP	COF/AuNPs	DPV	Chlorogenic acid	9.5 nM	[67]
	COF_DP-TFPPy_	Imine	DP, TFPPy	COF/AuNPs	DPV	Theophylline, caffeine	0.19, 0.076 μM	[68]
	COF_BD-Tp_	Imine	BD, Tp	COF/AuNPs	DPV	Bisphenol A	1 μM	[69]
	COF_BD-Tp_	Imine	BD, Tp	COF/PtNPs/MWCNTs	DPV	Tanshinol	0.018 μM	[22]
	COF_TAPB-DMTP_	Imine	TAPB, DMTP	COF/AuNPs/MWCNTs	DPV	Doxorubicin	16 nM	[70]
	COF_DHzDS-TFPB_	Imine	DHzDS, TFPB	COF/PtNPs@rGO	DPV	Furazolidone	0.23 μM	[71]
	COF_PDA-TFPPy_	Imine	PDA, TFPPy	COF/MWCNT-NH_2_/AuNPs	DPV	Dopamine, uric acid	0.21, 0.29 μM	[72]
	COF_TABQ-TQ_	Pyrazine	TABQ, TQ	/	DPV	Guanine, adenine	0.20, 0.33 μM	[73]
	COF_DABTZ-Tp_	Imine	DABTZ, Tp	COF/AChE	DPV	Methyl parathion, paraoxon, malathion	0.204, 0.794, 5.37 pg mL^−1^	[74]
	COF_TTA-Tp_	Imine	TTA, Tp	Post-carboxylation; COF/AuNPs	DPV	Gallic acid, uric acid	0.19, 0.25 μM	[75]
	COF_PDA-Tp_	Imine	PDA, Tp	2HP6@AuNPs@CP6@COF	DPV	Sodium picrate	1.7 nM	[76]
	COF_CTF-1_	Triazine	TPN	COF/SOD	i-t	Superoxide radicals	0.5 nM	[55]
	COF_BTH-DHNDA_	Imine	BTH, DHNDA	COF/AChE	CV	Carbaryl	0.16 μM	[77]
	COF_ETTA-TPA_	Imine	ETTA, TPA	COF/MPO-11, GOD	DPV	Glucose	4.97 μM	[78]
	COF_DBD-TFPB_	Imine	DBD, TFPB	COF/GOD, HRP, AChE	DPV	Glucose, H_2_O_2_, malathion	0.85 μM, 2.81 nM, 0.3 pg L^−1^	[14]
	COF_TAPB-DMTP_	Imine	TAPB, DMTP	Co_3_O_4_@COF	DPV	Tert-butylhydroquinone	0.02 μM	[23]
	COF_TAPB-DMTP_	Imine	TAPB, DMTP	FeNi@COF	DPV	Gallic acid	1.3 nM	[79]
	COF_TAPB-DMTP_	Imine	TAPB, DMTP	CuO nanorods@COF	DPV	Dopamine	0.023 μM	[80]
	COF_TAPP-TFB_	Imine	TAPP, TFB	COF/Fe^2+^	DPV	H_2_O_2_	2.06 nM	[81]
	COF_TAPB-DMTP_	Imine	TAPB, DMTP	COF/*β*-CD polymers/Pd^2+^	DPV	Norfloxacin	0.031 μM	[82]
	COF_Thi-TFPB_	Imine	Thi, TFPB	COF/NH_2_-CNTs	DPV	Ascorbic acid	17.68 μM	[83]
	COF_Thi-TFPB_	Imine	Thi, TFPB	COF/3D-MC	DPV	Riboflavin	44 nM	[84]
	COF_TTA-DHTA_	Imine	TTA, DHTA	COF/GOD	DPV	H_2_O_2_, glucose	1.70, 0.18 μM	[85]
	COF_ETTA-TPA_	Imine	ETTA, TPA	COF/Fc	DPV	H_2_O_2_	0.33 μM	[13]
	COF-LZU1	Imine	PDA, TFB	AgNPs/COF	DPV	Bisphenol A, bisphenol S	0.15, 0.15 μM	[86]
Ions Sensors	COF_TAPB-DMTP_	Imine	TAPB, DMTP	/	DPASV	Pb^2+^	1.9 nM	[52]
	COF_MA-TPA_	Imine	MA, TPA	Fe_3_O_4_ NPs@COF/bismuth film	SWASV	Pb^2+^	0.95 nM	[87]
	COF_TAPB-Dva_	Imine	TAPB, Dva	TTC post-modification; graphene	SWASV	Cd^2+^, Pb^2+^, Cu^2+^, Hg^2+^	0.3, 0.2, 0.2, 1.1 μg L^−1^	[53]
	COF_TTA-BTT_	Imine	TTA, BTT	/	DPSV	Hg^2+^	0.18 nM	[88]
	COF_MELE-BTDD_	Imine	MELE, BTDD	/	SWASV	Cd^2+^, Pb^2+^, Cu^2+^, Hg^2+^	4.74, 1.23, 1.14, 1.07 nM	[89]
	COF_MA-Tp_	Imine	MA, Tp	/	SWASV	Cd^2+^, Cu^2+^, Pb^2+^, Hg^2+^, Zn^2+^	0.92, 0.45, 0.31, 0.21, 0.53 nM	[90]
	COF_BTLP-1_	Imine	BDDH, TFB	COF/3D-MC	DPSV	Cd^2+^, Pb^2+^, Cu^2+^, Hg^2+^	12.3, 11.8, 18.6, 21.4 nM	[24]
	COF_TAPB-DMTP_	Imine	TAPB, DMTP	COF/TiO_2_-NH_2_	SWCSV	Mn^2+^	0.0283 nM	[91]
Immunosensors	COF_TAPB-DMTP_	Imine	TAPB, DMTP	COF/AuNPs/Ab/HRP	DPV	Cardiac troponin I	1.7 pg mL^−1^	[92]
	COF-LZU1	Imine	PDA, TFB	COF/AuNPs/Ab/TB	SWV	Cardiac troponin I	0.17 pg mL^−1^	[93]
	COF_BD-Tp_	Imine	BD, Tp	Fe_3_O_4_@COF/AuNPs/MB/Ab	DPV	Prostate specific antigen	30 fg mL^−1^	[94]
	COF-LZU1	Imine	PDA, TFB	COF/PtNPs/Ab	DPV	C-reactive protein	0.2 ng mL^−1^	[95]
	COF_TAPB-TPA_	Imine	TAPB, TPA	COF/AuNPs/Ab	DPV	Kidney injury molecule-1	2.0 fg mL^−1^	[96]
	COF_TAPB-DMTP_	Imine	TAPB, DMTP	COF/CuS NPs	DPV	Amyloid-*β* oligomer	0.4 pM	[97]
Aptasensors	COF_TAPB-TPA_	Imine	TAPB, TPA	aptamer/AuNPs@ZnFe_2_O_4_@COF	DPV	Norovirus	0.003 copies mL^−1^	[98]
	COF_Bpy-Tp_	Imine	Bpy, Tp	AuNPs@Ce-COF/aptamer	i-t	Zearalenone	0.389 pg mL^−1^	[99]
	COF_HHTP-BFc_	Imine	HHTP, BFc	COF/aptamer	DPV	Cardiac troponin I	2.6 fg mL^−1^	[100]
	COF_TAPB-DMTP_	Imine	TAPB, DMTP	COF/AuNPs/aptamer	EIS	Ciprofloxacin	2.34 fg mL^−1^	[101]
	COF_TAPB-DMTP_	Imine	TAPB, DMTP	COF/CNTs/aptamer	EIS	Atrazine	0.67 pg mL^−1^	[102]
	COF_MA-TFPPy_	Imine	MA, TFPPy	COF/aptamer	EIS	Ampicillin, enrofloxacin	0.04, 6.07 fg mL^−1^	[103]
	COF_TAPP-TPA_	Imine	TAPP, TPA	COF/aptamer	EIS	EGFR, MCF-7 cells	7.54 fg mL^−1^, 61 cells mL^−1^	[104]
	COF_CTF-1_	Triazine	TPN	Co-MOF@COF/aptamer	EIS	Ampicillin	0.217 fg mL^−1^	[56]

At present, most COFs applied in the EC sensing scope are still dominated by a traditional solvothermal synthetic method [105]. Figure 2A depicts the synthesis diagram of the first reported COF products with the triboronate ester linkage as an example [1]. Briefly, the reactant monomers, catalysts, and solvents are uniformly mixed in a Pyrex tube, possibly with the aid of sonication. Subsequently, this charged tube is flash-frozen with liquid nitrogen and degassed by a pump. Undergoing multiple freeze–pump–thaw cycles to remove oxygen, it was sealed under a vacuum. The crude products are produced at a suitable temperature for some time, and the precipitates are collected, washed, and dried to obtain the final products [47]. Although COFs structure at the macroscopic level can be regulated, this method involves tedious preparation procedures and harsh reaction conditions. Several simplified synthetic methods have been attempted, that are, for example, the preparation using a stainless-steel reactor lined with a Teflon vessel [2, 83, 88, 106], and direct polycondensation under ambient conditions without vacuuming and heating processes [60, 92]. Certainly, exploring facile and green synthetic strategies is a new trend in manufacturing COFs-based EC sensors, such as water-mediated, solid-phase, vapor-assisted, hydrothermal, and micelle-assisted synthesis approaches [107].

## COFs in EC Sensing

Crystalline COFs exhibit prominent advantages in achieving high-performance EC sensing compared with traditional covalent polymers and quite similar MOFs [2–5, 30, 32, 108–111]. First, COF networks built by *π*-stacking or extended *π*-conjugated backbones allow fast charge transport along the self-assembled molecular channel and show modest electrical conductivity. Second, the generally high specific surface area endows their high-load capacity of EC-active substances and a large electrode active area. Third, their macrostructures and nanosized pores can be tailored to accommodate specific guests via the non-covalent interaction (i.e., hydrogen bonds, *π*–*π*, hydrophobic, and electrostatic interactions), facilitating selective identification and enrichment of target molecules. Fourth, electroactive COFs with given redox patterns are utilized to develop accurate ratiometric EC sensing. Fifth, COFs with good flexibility are well distributed on the electrode surface without the assistance of membrane forming-reagents (e.g., Nafion and chitosan). Last but not least, COFs typically exhibit low toxicity, good biocompatibility, as well as high chemical and thermal stability, benefiting the development of repeatable and reproducible EC sensors, especially for the usages in biosensing. The relevant advances in COFs-based EC sensors including electrode materials, EC methods, analytes, and the limit of detection (LOD) are summarized in Table 2, and several representative ones are described in detail below.

### Chemical Sensors

Chemical sensors are involved in all aspects of life, including physiological check, drug evaluation, food testing, environmental monitoring, etc. [112]. Most chemical molecules have their own characteristic EC redox activities, making them easy and convenient to be directly recognized according to their electro-redox attributes. Thus, versatile sensors by means of EC technologies, especially differential pulse voltammetry (DPV), square wave voltammetry (SWV), and amperometry (*i-t*), have been widely designed for chemical sensing because of their superiorities of sensitivity, portability, automation, and low price [83, 85]. Importantly, highly adaptable structural and functional design, specific recognition, electroactivity, unique catalytic capabilities, as well as inherent natures including high specific surface areas, ordered channels, and acceptable stability, make COFs promising electrode substrates for chemical detection. Thanks to the ample examples of chemical sensors, in this section, these improvement strategies of COFs-based analysis are further classified into the following four parts according to the improvement strategies.

#### For Good Electroconductivity

Despite these advantages, current COFs as electrode materials are still limited due to their poor affinity and conductivity. Hybridization with carbon materials might be the simplest and most economical way to achieve conductivity improvement [113]. Carbon nanotubes (CNTs) contain *sp*^2^ hybridized carbon atoms in a graphite sheet-like manner, owning large delocalized *π* orbitals and high electrical conductivity [60]. Further, mainly through *π*–*π* stacking, the conjugated COFs can be non-covalently assembled on the outer surface of CNTs. For instance, following an one-pot synthesis at a mild reaction condition, COF_TAPB-TPA_ was immobilized in situ on an NH_2_–CNTs matrix with the building monomers of 1,3,5-tris(4-aminophenyl)benzene (TAPB) and terephthaldicarboxaldehyde (TPA). The resultant COF_TAPB-TPA_/NH_2_–CNTs presented nice conductivity and a large specific surface area, enabling the fabrication of a furazolidone EC sensor with a low LOD of 77.5 nM and acceptable recoveries ranged from 87.8 to 126.9% [60]. A similar work reported the growth of *β*-ketoenamine-linked COF_BTH-Tp_ on NH_2_–CNTs by dehydration condensation between benzene-1,3,5-tricarbohydrazide (BTH) and 1,3,5-triformylphloroglucinol (Tp). Based on the composite, an EC sensor displayed excellent performances for nitrofural detection with a LOD of 3.2 nM [61]. Li et al. proposed an ultrasonic-mediated method for the self-assembly of COF_TAPB-Dva_ (Dva: 2,5-divinylterephthalaldehyde)@graphitized multi-walled CNTs (MWCNTs) via the *π*–*π* conjugation effect. This silkworm-cocoon-like nanohybrid was further employed for the reliable detection of diuron in food samples, achieving a LOD of 0.08 μM and satisfactory recoveries from 96.40 to 103.20% [62]. Besides, conductive polymers can serve as conductive carriers for the in-situ loading of COFs. Using p-aminobenzoyl hydrazide (ABH) and 2,4,6-tris-(4-formylphenoxy)-1,3,5-triazine (TFPOT) as the monomers, Pan et al. accomplished a facile one-pot approach for in-situ growth of COF_ABH-TFPOT_ on the polyaniline, which amplified the response signal of sulfamethoxazole (SMX). This COFs-based EC sensor displayed a broad linear range (1–450 μM) and a low LOD (0.107 μM) for SMX, and was used for reliable testing of environmental water samples [65].

**Fig. 3:**
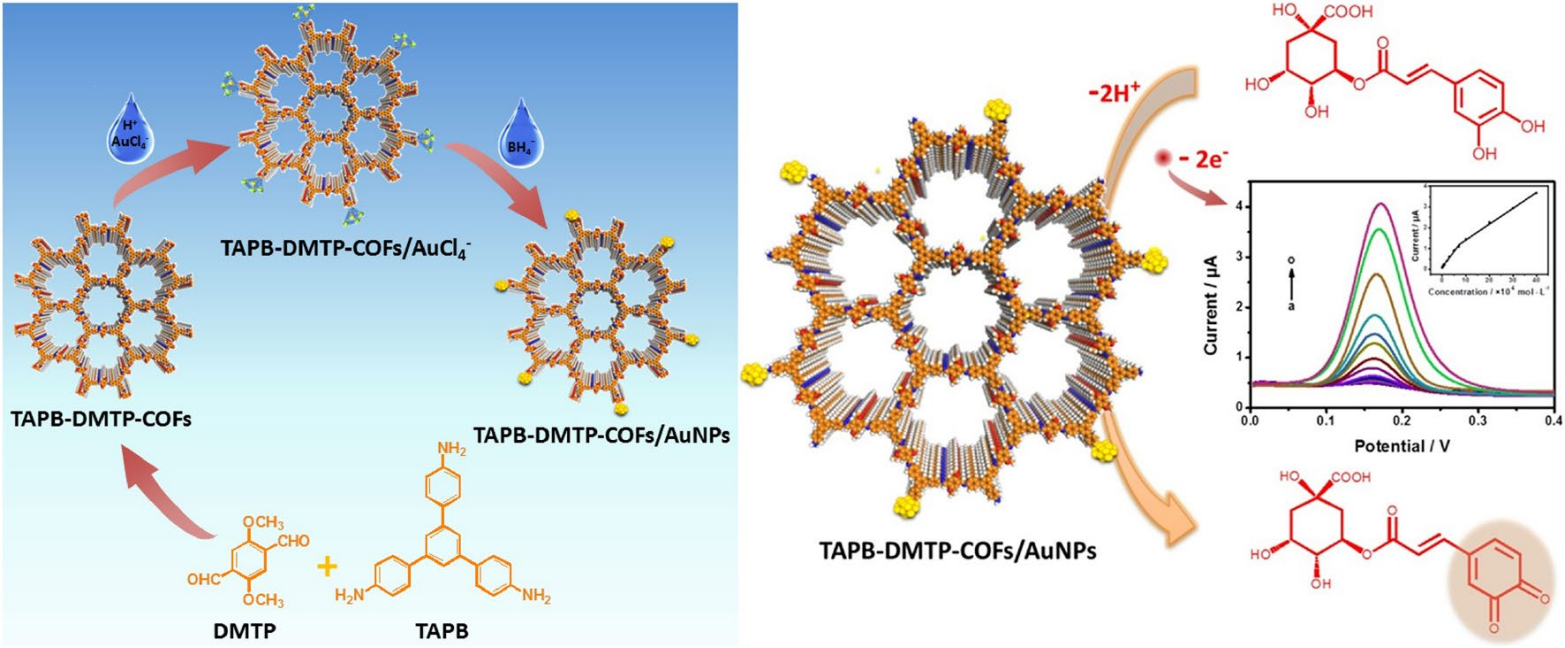
Schematic diagrams of the synthesis route for COF_TAPB-DMTP_/AuNPs and the resulting EC sensing for chlorogenic acid. Reproduced with permission from Ref. [67]. Copyright (2018) Elsevier

Nobel metal nanoparticles (NPs), known for excellence in conductivity, biocompatibility, and catalytic activity [66, 67], can be controllably grown and confined within the regular pore channels of COFs in a well-dispersed manner, thus fully exploiting the capabilities of COFs. For example, Lu and co-workers prepared COF_TAPB-TPA_/AuNPs via an in-situ growth method for enrofloxacin (ENR) detection. These loaded AuNPs-assisted redox reactions by lowering the overpotential, stabilizing reversibility, and accelerating charge transfer, thereby achieving a low LOD of 0.041 μM and good recoveries of 96.7–102.2% [66]. Similarly, Zhang and her colleagues fabricated an active and repeatable EC sensor for chlorogenic acid measurement based on COFs-supported AuNPs. In this work, AuNPs were prepared by *in-situ* chemical reduction and landed on the COF_TAPB-DMTP_ (DMTP: 2,5-dimethoxyterephaldehyde) surface due to the firm electrostatic absorption of the remaining –NH_2_ of COFs (Fig. 3) [67]. In another work, Guan et al*.* obtained a new COF by a Schiff-base reaction between 2,6-diaminopyridine (DP) and 4,4′,4″,4‴-(pyrene-1,3,6,8-tetrayl)tetrabenzaldehyde (TFPPy) through a tube oven heating program. Further compounded with AuNPs, the resultant COF_DP-TFPPy_/AuNPs were directly utilized for the fabrication of a sensitive EC sensor to evaluate theophylline and caffeine in compound paracetamol capsules and black tea samples, and the LODs were 0.19 and 0.076 μM, respectively [68].

The synergy of multiple conductive species usually contributes to better EC activity. For instance, Zhang et al. synthesized the spherical COF_BD-Tp_ (BD: benzidine) by a facile solution-phase method and loaded PtNPs by in-situ chemical reduction of H_6_PtCl_4_. After further integration of MWCNTs, the resultant ternary composites exhibited outstanding catalytic performance and electrical conductivity, affording effective drug sensing of tanshinol with a sensitivity up to 10.089 μA mM^−1^ and a LOD down to 0.018 μM [22]. Zhao and her colleagues developed an electrode matrix of COF_TAPB-DMTP_ co-decorated with AuNPs and MWCNTs. This dual conductive species-supported COFs sensor exhibited excellent stability, reproducibility, and selectivity for doxorubicin detection with a low LOD of 16 nM [70]. Chen et al. fabricated a paper-based EC sensor for the furazolidone detection based on the nanocomposites of PtNPs/COF_DHzDS-TFPB_ (DHzDS: 2,5-bis (3-(ethyl thiol) propoxy) *p*-benzoyl hydrazine; TFPB: 1,3,5-tris(*p*-formylphenyl) benzene)@reduced graphene oxide (rGO) [71]. In addition, Guan et al. established an EC sensing platform based on the composite of COF_PDA-TFPPy_, MWCNTs, and AuNPs. In this work, COF_PDA-TFPPy_ was prepared by aminaldehyde condensation between 1,4-phenylenediamine (PDA) and TFPPy. The synergy of two EC active substances endowed this COFs-based biosensor with high conductivity, extraordinary stability, and large specific surface area, thus achieving simultaneous detection of uric acid (UA) and dopamine (DA) with LODs of 0.29 and 0.21 μM, respectively [72].

Additionally, conductive COFs can be built directly for chemical sensors. For example, Pan et al. prepared COF_TABQ-TQ_ via a condensation reaction of tetraaminobenzoquinone (TABQ) and triquinoyl (TQ). This nitrogen-rich COF presented high crystallinity and good electrical conductivity (6.06 × 10^−3^ S cm^−1^), which was directly employed for the simultaneous analysis of guanine and adenine. The LODs were as low as 0.20 and 0.33 μM, respectively [73]. Wei and co-workers developed an interfacial perturbation growth method to obtain an ultrathin (≈ 1.95 mm) nitrogen and sulfur-rich bithiazole-based 2D-COF nanosheets (NSs) using the monomers of 2,2′-diamino-4,4′-bithiazole (DABTZ) and Tp. Owing to their remarkable electrical conductivity and abundant edge unsaturated sites, a high-performance biosensor was fabricated with acetylcholinesterase (AChE) as the biometric element for effective monitoring of organophosphorus pesticides [74].

#### For Enhanced Hydrophilicity

**Fig. 4:**
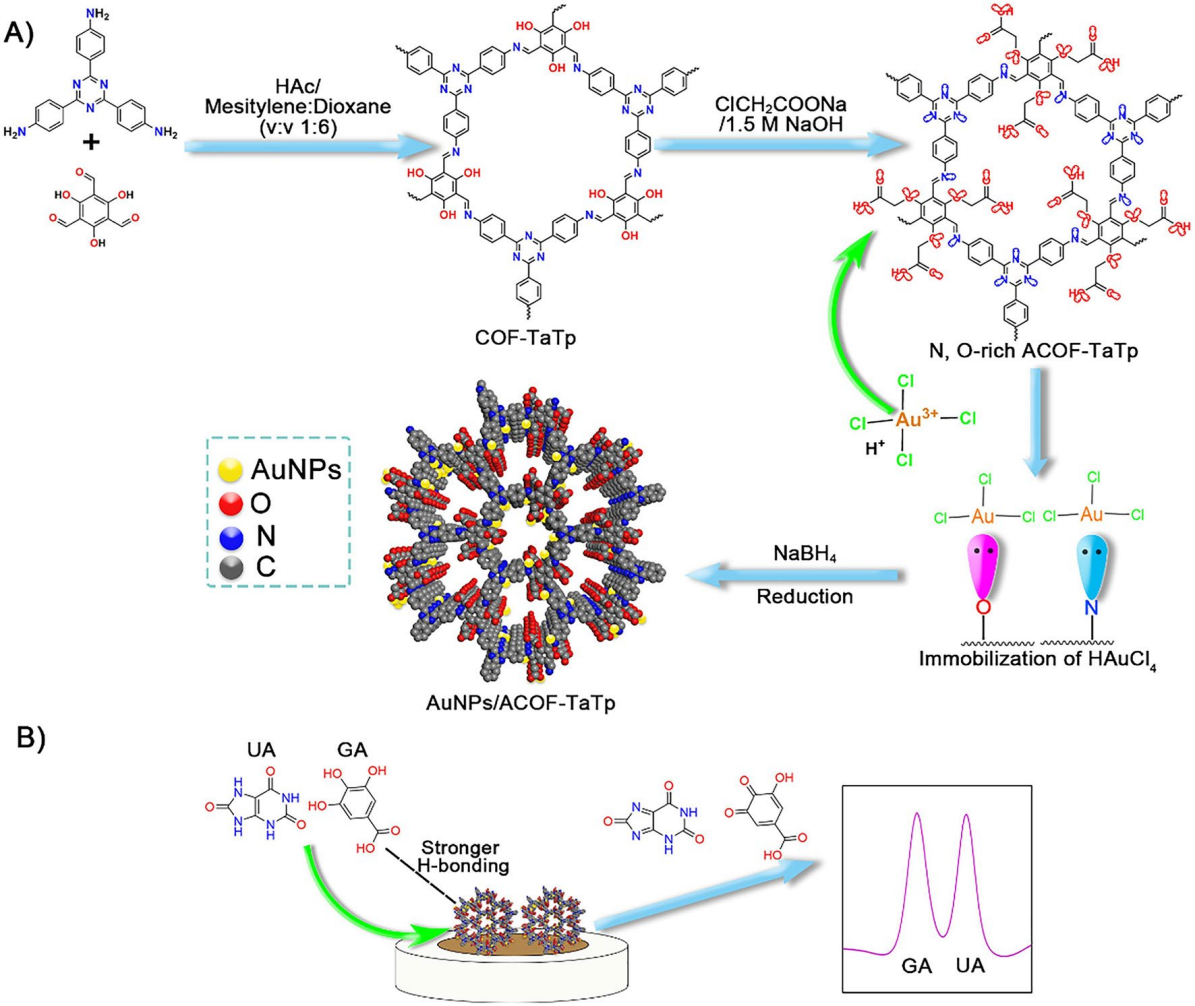
Schematic illustrations of the synthesis route for hydrophilic COF_TTA-Tp_/AuNPs and the resulting simultaneous EC sensing of GA and UA. Reproduced with permission from Ref. [75]. Copyright (2021) Elsevier

Since COFs are mostly composed of hydrophobic units, their poor hydrophilicity hinders efficient dispersion and causes weak contact between substances and reaction centers, inevitably affecting their EC activity and stability. To solve this problem, Lin’s group first prepared N, O-rich COF_TTA-Tp_ through a condensation reaction of 4,4′,4″-(1,3,5-triazine-2,4,6-triyl) trianiline (TTA) and Tp. Post-carboxylation was adopted for enhanced hydrophilicity, supplying sufficient electron donors of O and N heteroatoms. Then, HAuCl_4_ was attached and reduced in situ to anchor highly dispersed AuNPs. Meanwhile, the decorated –COOH showed a greater affinity for gallic acid (GA) than for UA through hydrogen bonding, resulting in their oxidation peaks being separated on the sensor. Accordingly, the constructed sensor realized simultaneous analysis of GA and UA, displaying wide linear responses (1–175 μM; 1–150 μM) and low LODs (0.19 and 0.25 μM), respectively (Fig. 4) [75]. In another work, AuNPs were immobilized on COF_PDA-Tp_ via supramolecular host–guest recognition of pillar [n]arenes. Specifically, macrocyclic hosts of dihydroxylatopillar [6]arene (2HP6) decorated AuNPs (2HP6@AuNPs) were prepared via in-situ reduction. Next, 2HP6@AuNPs were recognized on the cationic pillar [6]arene (CP6)@COF_PDA-Tp_. The final heterogeneous composites of 2HP6@AuNPs@CP6@COF_PDA-Tp_ with enhanced hydrophilicity were utilized for rapid EC monitoring of sodium picrate by virtue of the electrocatalysis of AuNPs, the recognition and enrichment of 2HP6 and CP6, as well as the outstanding supporting of COF_PDA-Tp_ [76].

#### For High Catalytic Performance

Enzymatic EC biosensors have received special attention because of the high efficiency and specificity of enzymatic reactions [14]. Nevertheless, native enzymes are vulnerable and prone to aggregation and inactivation during the construction and storage of sensors. Thus, immobilizing enzymes on COFs-supported matrices makes it possible to act synergically for both properties and advantages, sufficiently maintaining bioactivity, and distribution of enzymes. Yildirim et al. synthesized 2D triazine-based COF_CTF-1_ through cyclic trimerization using a single terephthalonitrile (TPN) monomer. Based on COF_CTF-1_ as the enzymatic support and superoxide dismutase (SOD) as the recognition element, an EC biosensor enabled the inspection of superoxide radicals in clinical samples with a 0.5 nM detection limit [55]. Xiao et al. covalently coupled AChE with the N, O-rich COF_BTH-DHNDA_ (DHNDA: 2,6-dialdehyde-1,5-dihydroxynaphthalene). Further using anionic [Fe(CN)_6_]^3−/4−^ as the sensing indicator, a turn-off EC biosensor based on a flexible carbon paper electrode enabled the carbaryl detection with a low LOD of 0.16 μM [77]. Wang et al. utilized the dual-pore COF_ETTA-TPA_ (ETTA: 4,4′,4″,4‴-(ethane-1,1,2,2-tetrayl) tetraaniline) to carry double enzymes of microperoxidase-11 (MPO-11) and glucose oxidase (GOD) for ratiometric EC biosensing of glucose, and the LOD was down to 4.97 μM. In this work, through pore encapsulation and hydrogen bonding, the two enzymes were well supported in different-sized pores of COF_ETTA-TPA_, further allowing their firm attachments onto the electrode surface [78].

**Fig. 5:**
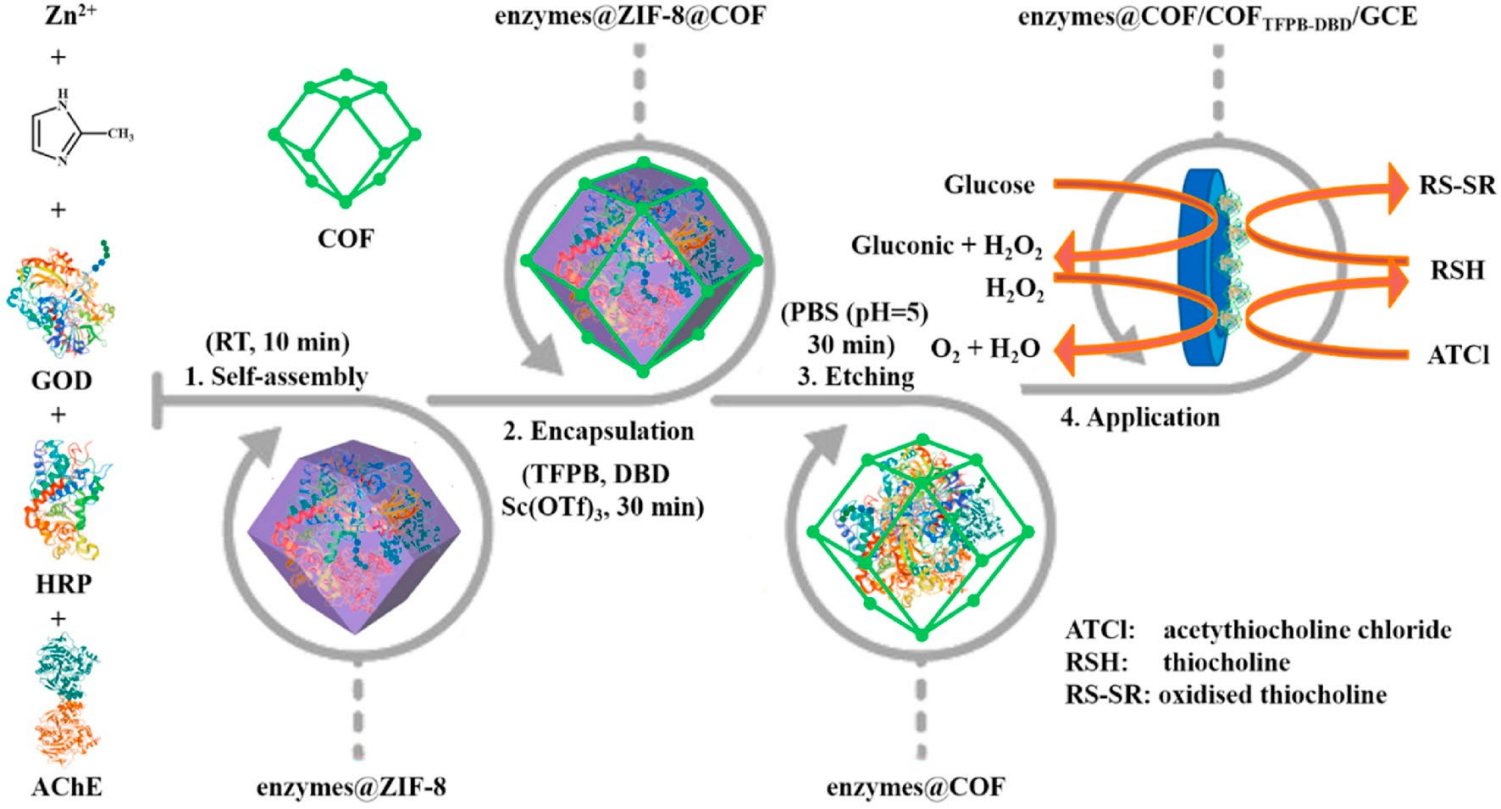
Schematic diagrams of the synthesis and usage of a multienzyme microcapsule. Reproduced with permission from Ref. [14]. Copyright (2021) Elsevier

Although COFs can indeed protect enzymes from harsh conditions, the micropores of COFs restrict the free conformation of enzymes, which inevitably affects their catalytic activity. To improve the freedom of the enzyme configuration, Liang et al. designed a multienzyme microcapsule with a COF shell and a 600 nm-sized cavity to encapsulate native enzymes. Specifically, three model enzymes of AChE, horseradish peroxidase (HRP), and GOD were first loaded into zeolitic imidazolate framework-8 (ZIF-8). Subsequently, a robust COF_DBD-TFPB_ shell was grown in situ outside the enzymes@ZIF-8 with 4,4′-diaminobiphenyl-2,2′-dicarboxylic acid (DBD) and TFPB via amine-aldehyde condensation. Ultimately, ZIF-8 was etched away to form a cavity loaded with multienzymes capable of free conformation. In comparison with those enzymes roughly stacked on the electrode surface, the biosensor constructed with this multienzyme microcapsule displayed superior catalytic performances, and the LODs achieved 0.85 μM, 2.81 nM, and 0.3 pg L^−1^ for glucose, H_2_O_2_, and malathion detection, respectively (Fig. 5) [14].

**Fig. 6:**
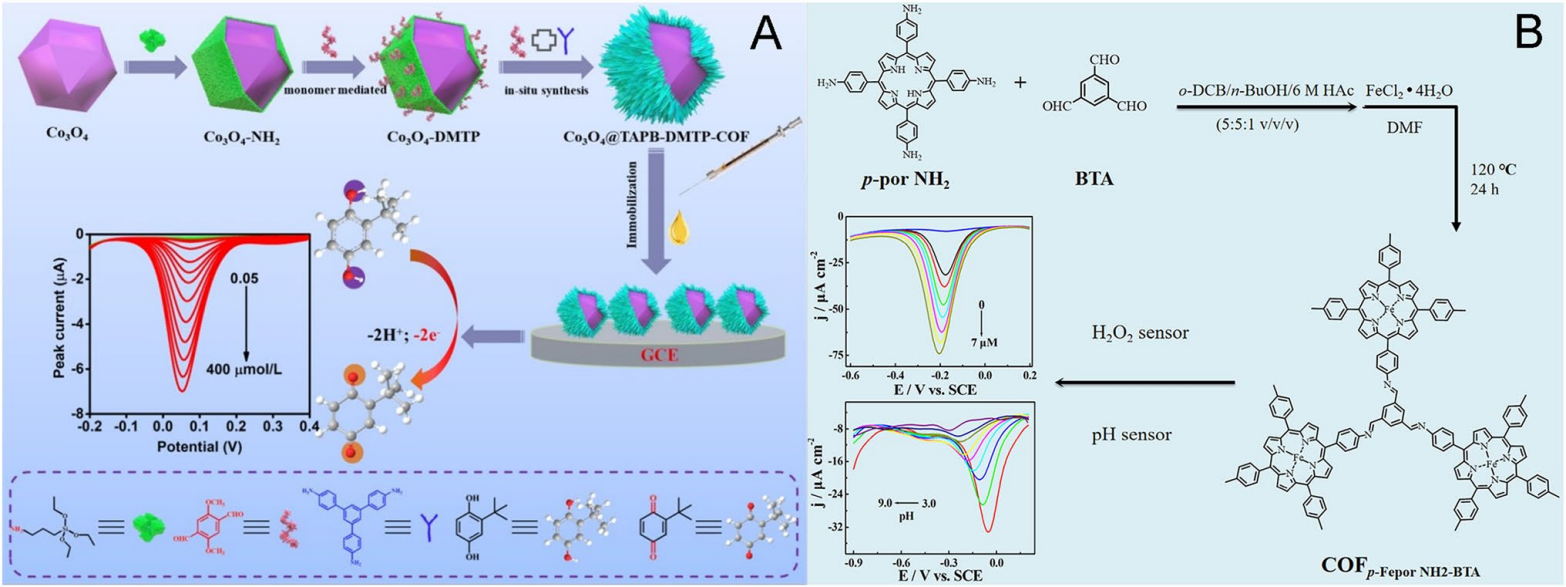
Schematic illustrations of the preparation routes and EC sensing applications of **A** Co_3_O_4_@COF_TAPB-DMTP_ and **B** COF_TAPP-TFB_/Fe^2+^. Reproduced with permission from Refs. [23, 81] Copyright (2021, 2020) Elsevier

Some transition metal oxides and complexes also possess mimic catalytic centers without being limited by the fragility of natural enzymes any more. To avoid aggregation, these centers can be doped into the COFs with good dispersion and unique catalytic activity for non-enzymatic EC sensing. For example, Chen’s group proposed a shape-tailored controlled assembly method for growing COF_TAPB-DMTP_ on the Co_3_O_4_ dodecahedrons. The core–shell composite exhibited uniform size, ultrahigh effective surface area, and excellent thermochemical stability, thus affording the tert-butylhydroquinone (TBHQ) EC sensing with a LOD as low as 0.02 μM. In this architecture, the Co_3_O_4_ core guaranteed the superior electronic and catalytic attributes, and 2D COF_TAPB-DMTP_ with multilayered columnar channels facilitated the facile shuttling of TBHQ toward the active Co^3+^ sites (Fig. 6A) [23]. In addition, Zha et al. synthesized a core–shell structured FeNi@COF_TAPB-DMTP_ nanocomposite with outstanding EC oxidation response toward GA and realized its sensing accordingly [79]. This group also prepared a similar CuO nanorods@COF_TAPB-DMTP_ core–shell composite for DA detection with a LOD of 0.023 μM [80].

Iron-porphyrin with Fe^3+^/Fe^2+^ electroactive centers exhibits peroxidase biomimetic activity, which can catalyze many EC reactions, such as the reduction of O_2_, H_2_O_2_, and phenolic oxides. For instance, Xie et al. prepared an iron-porphyrin-based COF with monomers of 1,3,5-triformylbenzene (TFB) and 5,10,15,20-tetrakis (4-aminophenyl)-21H,23H-porphine (TAPP) via an aldehyde-ammonia reaction. After the post-chelation with Fe^2+^, the resultant COF_TAPP-TFB_/Fe^2+^ served as a mimic peroxidase with remarkable EC redox and proton activity, enabling enzyme-free evaluation of H_2_O_2_ and pH (Fig. 6B) [81]. Chu et al. prepared three 2D metalloporphyrin COFs by altering the central metal atoms of Fe, Mn, and Cu. The authors demonstrated that the Fe porphyrin-COF/GCE sensor displayed the best electrocatalytic performance in the EC sensing of butylated hydroxy anisole [114]. In addition to porphyrin, some metal ions can also act as catalysts directly. Zhang et al*.* functionalized COF_TAPB-DMTP_ with *β*-cyclodextrin (*β*-CD) porous polymers and the catalytic Pd^2+^ element to fabricate a non-enzyme EC sensor for norfloxacin drug checking, showing two linear ranges of 0.08–7.0 μM and 7.0–100.0 μM with a LOD of 0.031 μM [82].

#### For Accurate Sensing Strategy

**Fig. 7:**
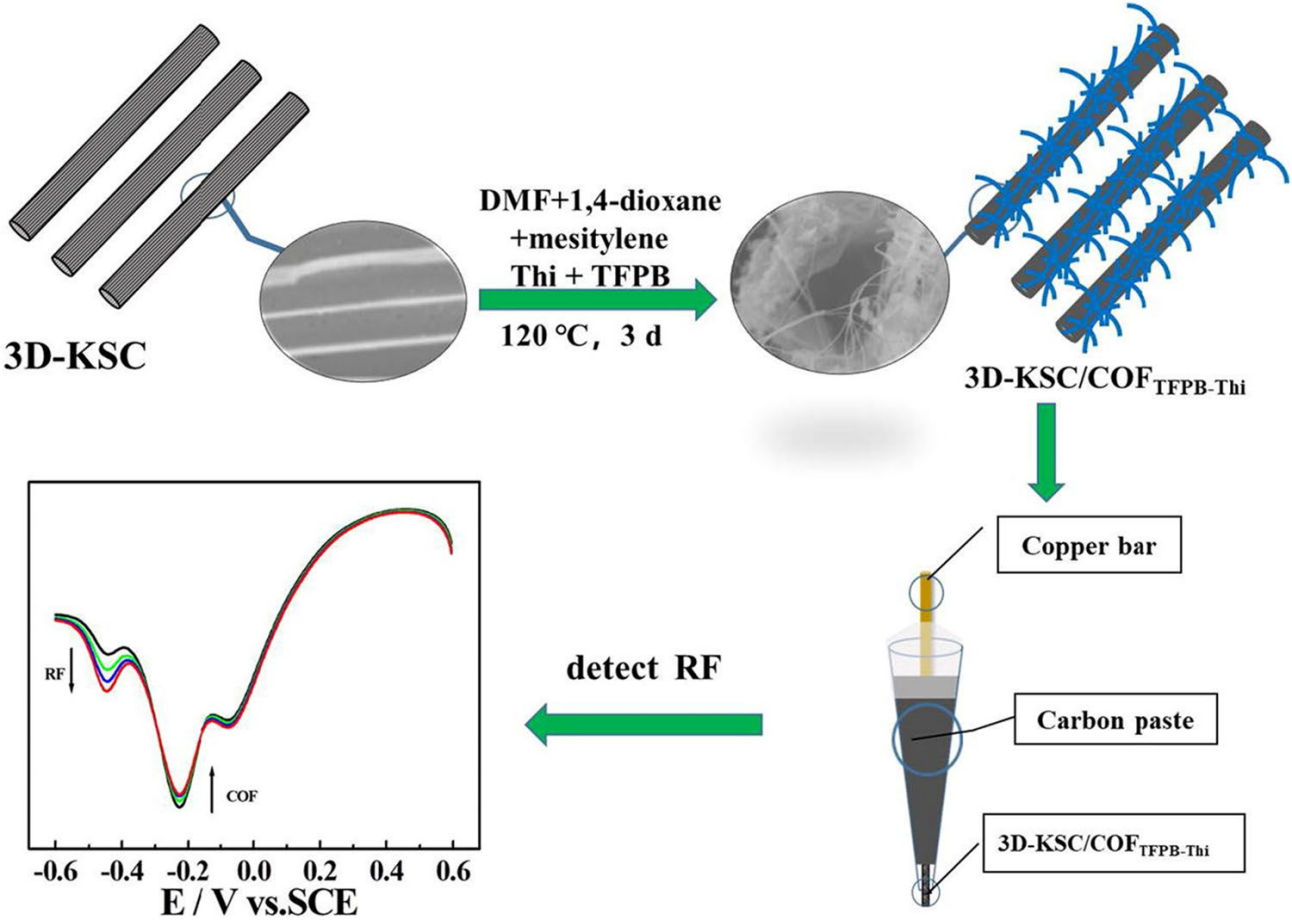
The synthesis process of 3D-MC/COF_Thi-TFPB_ and the fabrication of carbon paste electrode for ratiometric EC sensing of RF. Reproduced with permission from Ref. [84]. Copyright (2022) Elsevier

Ratiometric EC sensors based on two electrical signals are considered a significant advance due to their self-calibration, which can effectively avoid the errors caused by environmental or human factors, ensuring accurate target quantification [115]. It is exciting to note that the multiple redox peaks derived from electroactive COFs can directly serve as the response or reference signals for ratiometric sensing. For example, 2D COF_Thi-TFPB_ was synthesized from thionine (Thi) and TFPB via dehydration condensation and was further enwound by CNTs. With the improved stability and dispersibility, the COFs-supported CNTs exhibited prominent catalytic activity toward the oxidation of ascorbic acid (AA). Meanwhile, a pair of redox peaks assigned to electroactive COF_Thi-TFPB_ were inert to AA. As a result, a ratiometric strategy using the reference EC signal of COF_Thi-TFPB_ allowed for accurate AA sensing with a low LOD of 17.68 μM [83]. This group also grew COF_Thi-TFPB_ vertically on 3D macroporous carbon (3D-MC) to fabricate a carbon paste electrode for dual-signal ratiometric EC assays of riboflavin (RF) with a LOD as low as 44 nM. In this work, target RF molecules were first oxidized at + 0.6 V, and the partially formed oxidized RF (RF_ox_) further oxidized COF_TFPB-Thi_ (COF_ox_). During the negative potential scanning (+ 0.6 ~ − 0.6 V), the reduction pattern of the composite remained unchanged at − 0.23 V, whereas COF_ox_ and RF_ox_ were successively reduced at − 0.08 and − 0.45 V. Therefore, both the ratio results of *j*−0.08/*j*−0.23 V and *j*−0.45/*j*−0.23 V can be considered as the response signals, which complemented each other and made the quantification more accurate and reliable (Fig. 7) [84]. In another work, COF_TTA-DHTA_ prepared via aminaldehyde condensation between TTA and 2,5-dihydroxy terethaldehyde (DHTA) was also discovered with multiple redox-active states. Using COF_TTA-DHTA_ as the active material for H_2_O_2_ electrocatalysis, the reduction responses at − 0.3 V and − 0.5 V were gradually increased with the continuous addition of H_2_O_2_, while the reduction peak at 0.3 V was nearly kept constant. Thus, both *j*−0.3 /*j*+0.3 V and *j*−0.5/*j*+0.3 V can serve as ratiometric results for H_2_O_2_ sensing [85].

Additionally, extra electroactive molecules can be introduced into the COFs-based EC system to accomplish the ratiometric analysis. For example, ferrocene (Fc) dicarboxylic acid molecules were directly introduced into the synthetic process of COF_ETTA-TPA_ nanospheres. The enwrapped Fc molecules not only increased the interlayer distance of COF_ETTA-TPA_ but also promoted the self-disproportionation of H_2_O_2_. Then, the produced O_2_ was electro-reduced by COF_ETTA-TPA_ at − 0.5 V. Meanwhile, the reduction response at + 0.45 V assigned to Fc molecules lowered with the increasing dosage of H_2_O_2_. Thus, an “on–off” nonenzymatic ratiometric EC platform enabled the H_2_O_2_ detection, showing a high sensitivity of 0.009 μM^−1^ and a low LOD of 0.33 μM [13]. Pang et al. electrodeposited AgNPs on a flexible carbon cloth and drop-coated COF-LZU1, which was prepared via the aminaldehyde condensation between PDA and TFB. Based on this, a stable EC response derived from AgNPs served as a standard signal, and COF-LZU1 enriched the targets, affording sensitive ratiometric responses toward bisphenol A (BPA) and bisphenol S (BPS) with a same LOD of 0.15 μM [86].

### Ions Sensors

Accurate and selective ions sensing is of great importance in both environmental and biological fields [20, 116]. Given the different electro-redox properties of heavy metal ions (HMIs), anodic stripping voltammetry (ASV) is attractive for HMIs sensing because of its high sensitivity, simplicity, rapidity, and ease of online monitoring [52, 53]. Although ASV can play to its strengths, common problems such as poor stability and scarce identification elements still limit the detection of trace HMIs. By virtue of rich and diversiform absorption sites, COFs have attracted increasing attention in decorating high-quality HMIs sensing surfaces. For example, COF_TAPB-DMTP_, together with graphite powder and paraffin oil, was applied to manufacture carbon paste electrodes as ECL sensors, which achieved a remarkable current response to Pb^2+^ [52]. In another work, the sensitivity toward Pb^2+^ was significantly improved by modifying a glassy carbon electrode with an in-situ electroplated bismuth film and the nanocomposite of melamine (MA)-based COFs/Fe_3_O_4_ NPs [87].

The presence of COFs greatly enlarges the electroactive surface area, and these edge-terminal amino groups can serve as adsorption sites. However, marginal sites that rely on incomplete condensation of COFs are far from sufficient, while post-synthetic modifications with special functional groups can provide enough absorption sites. For instance, hydrosulphonyl functionalized COF (COF-SH) was synthesized via a solvothermal method with TAPB and Dva, followed by trithiocyanuric acid (TTC) post-modification. Using this COF-SH as the electrode substrate, abundant S and N sites accumulated HMIs, affording their sensitive EC quantification. The LODs were down to 0.3, 0.2, 0.2, and 1.1 μg L^−1^ for Cd^2+^, Pb^2+^, Cu^2+^, and Hg^2+^ detection, respectively [53].

Moreover, to avoid laborious and time-consuming post-processing, the N- or S-rich monomers of benzo [1,2-b:3,4-b′:5,6-b″] trithiophene-2,5,8-tricarbaldehyde (BTT) and TTA were selected for building rod-like COF_TTA-BTT_ through one-step amine-aldehyde condensation. COF_TTA-BTT_ was further employed for sensitive EC sensing of Hg^2+^, showing a low LOD of 0.18 nM due to its significant enrichment [88]. Also, COF_MELE-BTDD_ with multiple active sites (N–S–N, –C=N) was prepared for the HMIs measurement by amine-aldehyde condensation of 2,5,8-triamino-s-heptazine (MELE) and 4,4′-(benzo[c] [1, 2, 5] thiadiazole-4,7-diyl)dibenzaldehyde (BTDD) [89]. The same group also synthesized lamellar COF_MA-Tp_ using monomers of MA and Tp, and each unit displayed six adsorption sites (–C=N, –NH_2_, –C=O) for the selective capture of HMIs [90].

In order to increase the solubility of large-sized COFs, controlled assembly of COF_BTLP-1_ on 3D-MC derived from kenaf stem was achieved by directly introducing 3D-MC into the amine-aldehyde polymerization process between 1,4-benzenedithiol-2,5-diamino-hydrochloride (BDDH) and TFB. The resultant COF_BTLP-1_/3D-MC composite contained regular transport channels and twelve adsorption sites for both the transfer and adsorption of HMIs, which was further exploited for simultaneous detection of HIMs [24]. Besides, COF_TAPB-DMTP_ was also selected for controllable growth on the surface of TiO_2_–NH_2_ via Schiff-base condensation. The nanocomposite was successfully employed for the determination of Mn^2+^ in Chinese liquor, and the LOD was as low as 0.0283 nM [91].

### Immunosensors

**Fig. 8:**
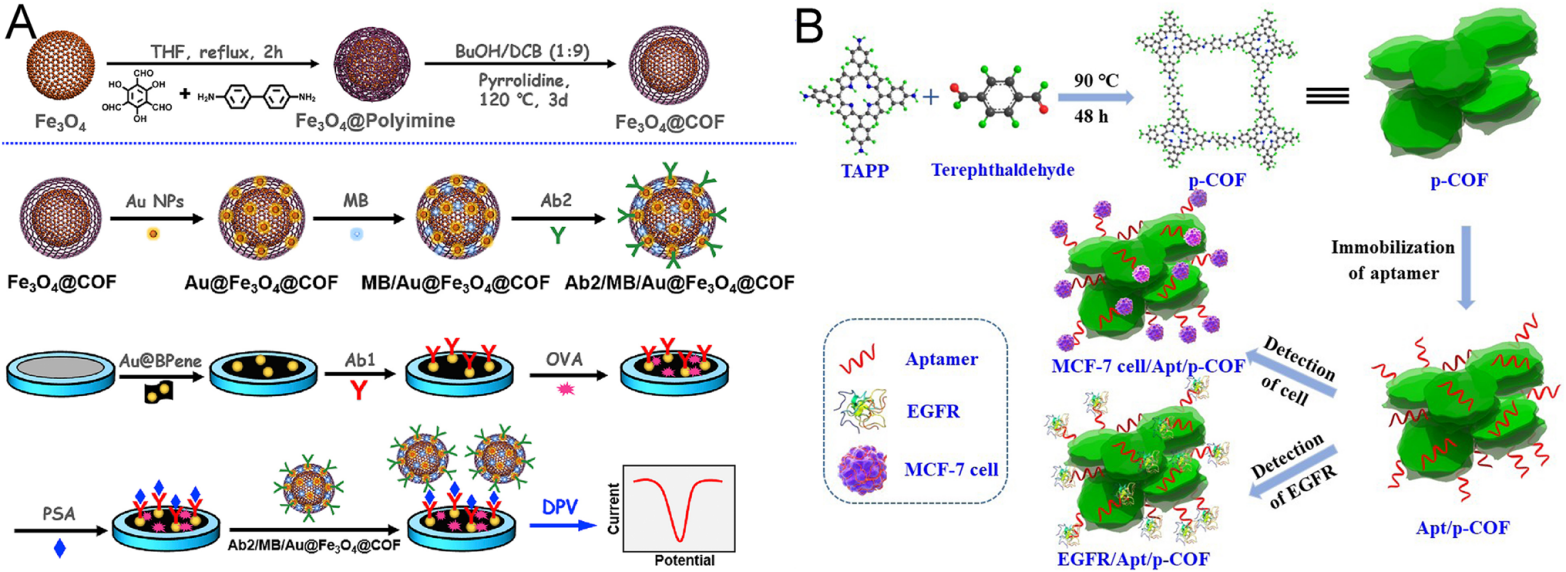
Schematic illustrations of **A** the preparation of Fe_3_O_4_@COF_BD-Tp_ and the construction of an EC immunosensor for PSA detection, and **B** the synthesis of COF_TAPP-TPA_ and the fabrication of an EC aptasensor for MCF-7 cells and EGFR sensing. Reproduced with permission from Refs. [94, 104] Copyright (2019) Elsevier

Immunosensors relying on the highly specific recognition between antibodies (Abs) and antigens are prominent tools for the effective measurement of biomarkers [21]. During the past decades, EC immunological detection has aroused considerable concern because of its high sensitivity, splendid selectivity, simple operation, rapid response, and low sample consumption [117]. Generally, sandwich-type immunosensing has been recognized as one of the most popular modes, which utilizes captured Abs for target identification and reporter Abs tagged with probes or triggers to indicate signal changes [20, 118]. Thus, signal labels are the key points for signal stability and amplification. Using the spherical COF_TAPB-DMTP_ as a carrier to sequentially load AuNPs, Abs, and HRP, a COF-supported signal probe was formed for the sandwiched EC immunosensing of cardiac troponin I (cTnI), a reliable biomarker of acute myocardial infarction, with a low LOD of 1.7 pg mL^−1^. Here, due to the enzymatic capacity of HRP, hydroquinone (HQ) was oxidized to benzoquinone (BQ) in the presence of H_2_O_2_, and BQ was then electro-reduced to output the amplified EC signals [92]. Also, COF-LZU1 was co-doped with AuNPs, Abs, and the electron mediator toluidine blue (TB) to behave as a signal probe. Further using polypyrrole-modified TiO_2_ NPs as the sensing substrate, a sandwich-type EC immunosensor realized the cTnI detection, exhibiting linearity ranging from 0.5 pg mL^−1^ to 10.0 ng mL^−1^ and a low LOD of 0.17 pg mL^−1^ [93]. Moreover, a magnetic COF_BD-Tp_ carried methylene blue (MB) for signal amplification. Briefly, magnetic COF_BD-Tp_ was synthesized by encapsulating Fe_3_O_4_ nanocrystals with an amorphous polyimine network through a Schiff-base reaction, which was further manipulated into crystalline imine-linked COFs under thermodynamic control. Through multi-noncovalent interactions, abundant MB molecules were incorporated into the macrocyclic supramolecular hosts of COF_BD-Tp_. Hence, using black phosphorene as a highly conductive matrix and this magnetic COFs-based probe for signal amplification, a sandwiched EC immunoassay realized the sensitive measurement of prostate specific antigen (PSA), an available serum biomarker of prostate tumors. The fabricated sensor achieved high analytical performance with a low LOD of 30 fg mL^−1^ (Fig. 8A) [94].

Another significant issue is to create a highly conductive, catalytic, and reusable sensing interface. The intrinsic properties of COFs make them favorable as an emerging class of electrode matrixes for supporting substances. In particular, 2D COFs with periodic layered arrays of *π* clouds can promote charge/carrier transport. For example, Liu et al. demonstrated the use of PtNPs-decorated COF-LZU1 as an EC platform for immunoassay. Meanwhile, AuNPs/MOFs (HKUST-1) complexes containing massive Cu^2+^ ions served as EC probes to show the expression of C-reactive protein (CRP), an effective indicator of infection, with a low LOD of 0.2 ng mL^−1^ [95]. Besides, Boyacıoğlu et al. fabricated a sandwich-typed EC immunosensor for kidney injury molecule-1 (kim-1) detection based on AuNPs/COF_TAPB-TPA_ as the electrode substrate and NiCo_2_S_4_@CeO_2_ microspheres as the signal amplification tags. The prepared immunosensor exhibited good specificity, desirable stability, and acceptable reproducibility with a LOD down to 2.00 fg mL^−1^ [96]. Chen et al. developed a signal on–off ratiometric EC immunosensor to detect Amyloid-*β* oligomer (A*β*O), a reliable biomarker for the early diagnosis of Alzheimer’s disease. In this work, ultrasmall CuS NPs-loaded COF_TAPB-DMTP_ acted as the electrode substrate to catalyze HQ oxidation for detectable signals. At the same time, electroactive Thi-decorated AuNPs served as another EC signal label. After A*β*O was introduced, the HQ signal decreased while the Thi signal increased, resulting in the ratios for the accurate A*β*O evaluation with a low LOD of 0.4 pM [97].

### Aptasensors

Aptamers represent synthetic single-stranded DNA or RNA oligonucleotides with unique 3D configurations, which can non-covalently bind to target molecules specifically [119, 120]. Aptasensors have been excitingly exploited by virtue of the advantages of aptamers, such as easy synthesis, low cost, good stability, and specific recognition ability. It is worth noting that the attributes of COFs guarantee the high loading capacity of aptamers via multiple interaction forces, thus establishing effective EC aptasensing platforms [46].

Similar to the sandwich-type immunosensor, a “peptide-target-aptamer” EC biosensor was constructed for norovirus detection based on Au@black phosphorous NSs@Ti_3_C_2_-MXene nanohybrids as the electrode substance and Au@ZnFe_2_O_4_@COF_TAPB-TPA_ as the magnetic tag. The redox probe TB can be further linked to the magnetic tags to output an increased EC signal. Thus, the biosensor achieved desirable selectivity, anti-interference, and stability with a low LOD of 0.003 copies mL^−1^_,_ and it was successfully utilized for norovirus detection in stool samples without complex pretreatment [98]. Moreover, aptasensor can directly utilize the high-impedance target biomass to hinder the electron and mass transfer, thereby causing the decreased EC signals in a non-label manner. For example, Chen and his colleagues prepared the AuNPs@Ce-COF_Bpy-Tp_ (Bpy: 2,2′-bipyridine-5,5′-diamine) nanocomposite as the electrode matrix for aptamer immobilization. The sensors achieved the signal-off determination of zearalenone (ZEN) toxin, showing a low LOD of 0.389 pg mL^−1^ and a recovery in the range of 93.0–104.7% [99]. In another work, Song et al. synthesized Fc-based COF_HHTP-BFc_ by connecting 2,3,6,7,10,11-hexahydroxytriphenylene (HHTP) and 1,1′-ferrocenediboronic acid (BFc) via boronate esters linkages, and its Fc moiety displayed remarkable voltammetric response. Then, a signal-on aptasensing strategy allowed for the label-free cTnI detection, because the affinity between cTnI and the specific aptamers drove away the aptamers from the COF_HHTP-BFc_ surface, recovering its EC response [100].

Electrochemical impedance spectroscopy (EIS), a simple, sensitive, label-free, and rapid method, has been widely used to collect signals generated by binding the analyte to the specific aptamer-functionalized transducer interface. For example, Zhu et al. prepared COF_TAPB-DMTP_/AuNPs and COF_TAPB-DMTP_/CNTs as the covered substrates, which had a strong non-covalent affinity toward the aptamers of ciprofloxacin (CIP) and atrazine. Then, two EIS aptasensor achieved the detection of CIP and atrazine, showing low LODs of 7.06 fM and 3.11 pM, respectively [101, 102]. Wang et al. prepared an imine-linked COF_MA-TFPPy_ by polycondensation of MA and TFPPy. COF_MA-TFPPy_ showed strong immobilization of aptamers and fast charge-carrier mobility because of its high specific surface area, large pore cavities, rich surface groups, and extended *π*-conjugated frameworks. As such, a COF_MA-TFPPy_-based EIS aptasensor allowed for sensitive determination of antibiotics, ampicillin (AMP) and ENR, yielding extremely low LODs of 0.04 fg mL^−1^ and 6.07 fg mL^−1^, respectively [103]. This group also synthesized 2D porphyrin-based COF_TAPP-TPA_. The highly conjugated NSs structure not only possessed improved EC activity but also facilitated the attachment of aptamers and biomolecules. The COF_TAPP-TPA_-based aptasensor displayed remarkable analytical performance for living Michigan Cancer Foundation-7 (MCF-7) cells and epidermal growth factor receptor (EGFR) (Fig. 8B) [104]. In addition, a novel nanoarchitecture of Co-MOF@COF_CTF-1_ was synthesized by directly introducing the as-prepared COF_CTF-1_ into the Co-MOF synthesis process. This multilayered Co-MOF@COF_CTF-1_ was used for EIS detection of AMP, one of the most frequently used *β*-lactam antibiotics, with an ultra-low LOD of 0.217 fg mL^−1^ [56].

## COFs in ECL Sensing

A surge of interest in COFs has emerged in the ECL realm, but ECL research on COFs is still in its infancy. The coreactant ECL mechanism describes an electro-triggered luminescence with the assistance of appropriate coreactants, which is the most common route because of its high efficiency and easy implementation. Taking the example of potassium persulfate (K_2_S_2_O_8_), a representative “reduction–oxidation” coreactant for ECL-active COFs, the ECL emitting route is described as the Reactions 1–4. Briefly, S_2_O_8_^2−^ is electro-reduced to produce the strong oxidant radical intermediate SO_4_^·−^. Meanwhile, ECL-active COFs undergo electro-reduction to form COFs^·−^. As a result, the radiative recombination of SO_4_^·−^ and COFs^·−^ generates high-energy COFs* to output ECL. To achieve high analytical sensitivity of ECL sensors, developing efficient ECL emitters and designing nanomaterials-based amplification strategies are the two main ways [31, 121–125], both of which can be realized by COFs.

1 $${\text{S}}_{2} {\text{O}}_{8}^{2 - } + e^{ - } \to {\text{SO}}_{4}^{2 - } + {\text{SO}}_{4}^{ \cdot - }$$

2 $${\text{COFs}} + e^{ - } \to {\text{COFs}}^{ \cdot - }$$

3 $${\text{COFs}}^{ \cdot - } + {\text{SO}}_{4}^{ \cdot - } \to {\text{COFs}}^{*} + {\text{SO}}_{4}^{2 - }$$

4 $${\text{COFs}}^{*} \to {\text{COFs}} + h\nu$$

Unlike MOFs with metal nodes that might extinguish the ECL [126–128], metal-free COFs seem to be more suitable as ECL luminophores [125, 129]. Likewise, their general properties, such as high specific surface area and porous structure, guarantee high loading capacity and easy mass transport. As such, COFs can be activated with strong ECL emission by constructing intra-reticular charge transfer (IRCT) using non-ECL active monomers [59, 129]. At the same time, the rigid framework can restrict the intramolecular rotations and vibrations of luminogens to achieve aggregation-induced emission (AIE) effects [130]. Additionally, pore confinement and enrichment of COFs are also exploited as reliable substrates for special and sensitive ECL sensing applications [57, 131]. Therefore, COFs show great potential for designing next-generation ECL sensing devices. In this section, the reported COFs used in the ECL field will be discusssed as ECL and non-ECL active ones.

### ECL Active COFs

**Fig. 9:**
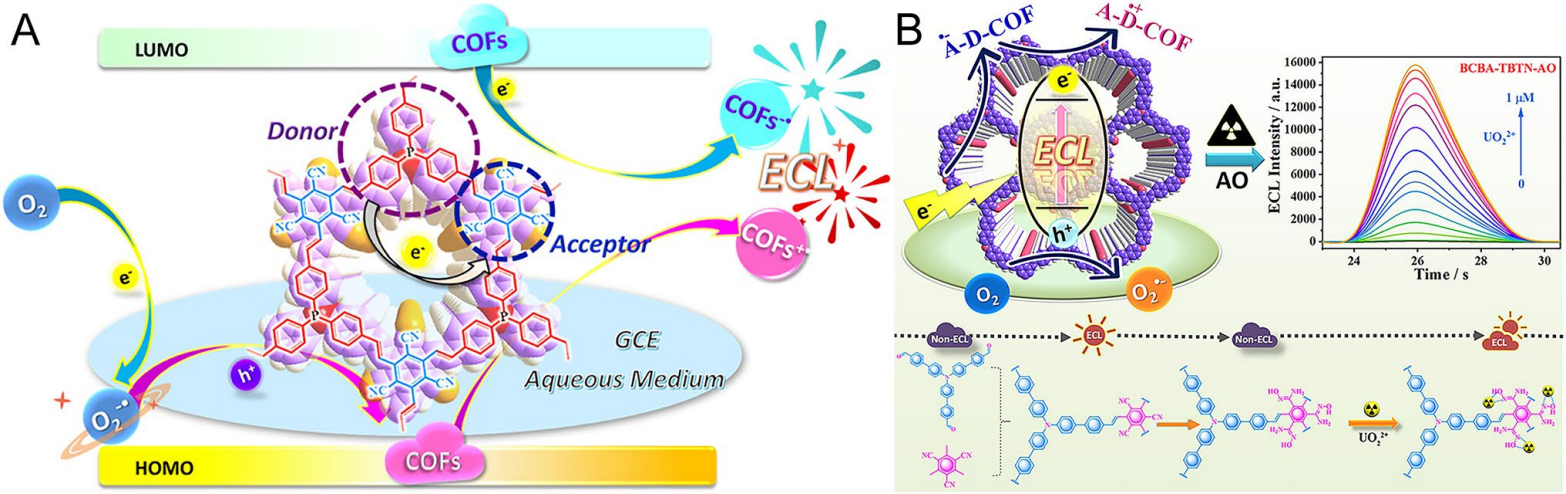
**A** Mechanism diagram of D-A fully conjugated COFs outputting ECL via IRCT. Reproduced with permission from Ref. [133]. Copyright (2021) American Chemical Society. **B** Schematic illustrations of the synthesis route for COF_TBTN-BCBA_ and the ECL sensing application for UO_2_^2+^. Reproduced with permission from Ref. [134]. Copyright (2021) American Chemical Society

ECL depends on the charge transfer between luminophores and co-reactive groups or molecules [20, 132]. Thus, designing functional blocks to modulate the charge-transfer behaviors of the long-range ordered COFs can activate effective ECL emission. It is a promising approach to achieve efficient IRCT by integrating electron-donor and acceptor units into a reticular skeleton via topology-templated conjugation. For example, Luo et al. designed a type of donor–acceptor (D–A) COFs with triazine and triphenylamine motifs as strong ECL emitters. A control COF with slight D-A contrast was also prepared by replacing the triazine units with analogous benzene ones. By comparison, the optimal D–A COF regulated by strong IRCT displayed a 123-fold increase in ECL. Experiments and density functional theory (DFT) calculations confirmed that the ECL behaviors were related to the crystallinity and protonation of the COFs, indicating the presence of IRCT between the D–A units. Further, the IRCT-mediated competitive oxidation mechanism afforded the decoding of the dual-peak ECL pattern of this D–A COF [129]. In another work, Qiu’s group reported a general strategy for assembling a series of olefin-linked D–A COFs as ECL emitters. These D–A COFs are composed of two key acceptor subunits of 2,4,6-trimethyl-1,3,5-triazine (TMT) and 2,4,6-trimethylpyridine-3,5-dicarbonitrile (DCTP) with C_3v_ and C_2v_ symmetry, respectively, exhibiting different ECL responses. Through increasing chain length and blocks conjugation, the ECL efficiency was boosted. Further, efficient IRCT allowed robust ECL output without the need for exogenous toxic coreactants [59]. Following this work, the same group also investigated highly aligned D–A COFs with olefin linkages by co-crystallizing four electron-rich molecules (i.e., benzaldehyde,4,4′4″-phosphinidynetris (BAP), 2,4,6-tris(4-formylphenyl)-1,3,5-triazine (TAPT), TFPB, and tris(4-formylphenyl)amine (TFPA)) and two electron-deficient molecules (i.e., TMT and 2,4,6-trimethylbenzene-1,3,5-tricarbonitrile (TBTN)) separately. Among these COFs, a tunable ECL was activated, and COF_BAP-TBTN_ displayed the maximum ECL efficiency (Ф_ECL_) of 32.1% in an aqueous solution with the coreactant of dissolved oxygen (O_2_). Quantum-chemical calculations suggested that the outstanding ECL performance was attributed to the reduced band gap and the good overlap of carriers within the excited COFs (Fig. 9A) [133]. Soon after, this group also regulated IRCT within two other olefin-linked COFs, assembled with an acceptor of TBTN and two donors of 4-[4-[3,5-bis[4-(4-formylphenyl)-phenyl]phenyl]phenyl]benzoic acid (DAFB) and 4-[4-[4-(4-formylphenyl)-*N*-[4-(4-formylphenyl)phenyl]anilino]phenyl]-benzaldehyde (BCBA), respectively. In a similar manner, the D-A couples-powered IRCT aroused the ECL emitting out of these non-ECL active monomers. With endogenetic O_2_ as the coreactant, COF_TBTN-BCBA_ achieved prominent Ф_ECL_ of 63.7% in a water medium, which was further employed for accurate ECL monitoring of UO_2_^2+^ with a proportionable ECL increase (Fig. 9B) [134]. Following similar design concepts, in addition, this group also synthesized a class of ECL available COFs by conjugating BTT as a key electron donor and three electron receptors of TBTN, DCTP, and TMT via C=C bonding [130].

AIE refers to the fact that a material emits intense luminescence in the aggregated state, but no or weak light in the solution state [135, 136]. Developing AIE emitters is meaningful because ECL luminophores in practical use are usually randomly aggregated on the electrode surface and easily cause ECL quenching [135–137]. As it is, the rigid skeleton of COFs can restrict intramolecular vibrations and rotations of the AIE luminogens via intralayer covalent bonds and interlayer non-covalent *π*-interactions, resulting in reduced nonradiative transitions and the enhanced AIE effects. For example, an AIE unit, 4,4′,4″,4‴-(ethene-1,1,2,2-tetrayl)tetrabenzaldehyde (ETB), was utilized as a key monomer to prepare three olefin-linked COFs. These COFs exhibited predominant framework-induced ECL in aqueous solution, far exceeding those of monomeric aggregated states, because the ordered framework not only promoted efficient charge transfer for an excited-state generation but also reduced the ETB rotation-caused nonradiative loss [138]. Li et al. designed an AIE-COF by a Schiff base reaction between TAPT and 4,4′-diamino-2,2′-bipyridine (DB), appearing to an excellent ECL performance in the presence of H_2_O_2_. Further coupled with Co_3_O_4_ nanozyme for amplifying the signal, a molecularly imprinted polymer (MIP) ECL biosensor was constructed for antibiotic chloramphenicol testing with a LOD of 0.118 pM [139]. In another work, [(1,3,5-triazine-2,4,6-triyl)tris(benzene-4,1-diyl)]triboronic acid (TTBT) was used to design an AIE-COF material via boric acid dehydration condensation, which was further employed for the construction of a MIP ECL biosensor [140]. Zhang et al. synthesized pyrene-based *sp*^2^ carbon-conjugated COF NSs by C=C polycondensation between 2,2′-(1,4-phenylene) diacetonitrile (PDAN) and TFPPy. In this structure, the AIE-luminogens of cyano-substituted phenylenevinylene and the pyrene luminophores were topologically connected together to reduce the aggregation-caused quenching effect. Further using Bu_4_NPF_6_ as the coreaction accelerator of the COF NSs/S_2_O_8_^2−^ ECL system, an ECL genosensor enabled the microRNA-21 detection with a low LOD of 46 aM via three cascaded Exo III-mediated recycling processes for signal amplification [141].

Although COFs have a high assembling capacity for ECL phosphors or motifs, the intrinsic poor conductivity still limits the ECL performance (generally < 10^−8^ S m^−1^). To address this issue, Zhang et al. designed a conductive COF with a fully *π*-conjugated planar structure by connecting HHTP and 2,3,6,7,10,11-hexaaminotriphenylene (HATP) via pyrazine linkages. The splendid ECL emission was achieved as a result of the superior electronic conductivity (3.11 × 10^−4^ S m^−1^), the luminophores directly as building blocks, and the porous structure that allows free diffusion of electrolyte. Based on COF_HHTP-HATP_ after prereduction electrolysis in S_2_O_8_^2−^ solution as an efficient solid-state ECL material, an “off–on” ECL platform was established for sensitive determination of thrombin through aptamer/target proximity binding-mediated 3D bipedal DNA walker for signal amplification [58]. Cui et al. prepared a fully-*π* conjugated COF_DMTP-TCPB_ with *sp*^2^ carbon-linkage via Knoevenagel polycondensation of DMTP and 1,3,5-tris(4-cynomethylphenyl)benzene (TCPB), exhibiting high ECL efficiency for alkaline phosphatase detection [142].

Additionally, various ECL-active COFs are springing up, accompanying their ECL sensing applications. For instance, Yang et al. designed a stable Ru-complex-based metal-COF using a classic ECL luminophore, tris(4,4′-dicarboxylicacid-2,2′-bipyridyl)ruthenium(II) (Ru(dcbpy)_3_^2+^), as a building motif. Owing to the topologically ordered and porous architectures, this Ru-MOF displayed strong ECL emitting and remarkable chemical stability for the microRNA-155 detection, achieving an ultralow LOD of 3.02 aM [143]. Song et al. demonstrated the synthesis of an aminal-linked COF using the luminescent monomers tetraphenylethylene (TPE) derivative and piperazine, which was further utilized to establish an ECL platform to selectively discriminate enantiomer of phenylalanine [144]. The same group also exploited this aminal-linked COF for glutathione (GSH) ECL assay with a LOD of 17 nM. In this work, target GSH can reduce the quencher of MnO_2_ NSs into Mn^2+^, restoring the ECL emission accordingly [145]. Lately, Qiu’s group designed structural isomerism of COFs with different ECL effects, which were further employed for the sensitive toxic As(V) detection with an ultralow LOD of 0.33 nM [146]. This group also constructed a COF-based host–guest system by guest molecular assembly with strong ECL emission and realized the nuclear contamination analysis of UO_2_^2+^ [147]. Besides, ECL potential tunable COFs are realized by changing the electron and spatial in the precursor, and the generated COF skeleton with reduced potential and increased ECL intensity was used for the selective ECL detection of lutetium ion, showing a LOD as low as 1.6 nM [148].

### Non-ECL Active COFs

In addition to being directly used as ECL emitters, COFs can also be made into microreactors to accumulate luminophores and coreactants, thus boosting ECL efficiency due to the confinement effect. For example, Zeng et al. loaded tris(2,2′-bipyridyl) ruthenium(II) (Ru(bpy)_3_^2+^) onto COF-LZU1, displaying increased nearly fivefold ECL intensity with a standard of classical Ru(bpy)_3_^2+^/tripropylamine (TPrA) system. In this work, COF-LZU1 can not only load massive Ru(bpy)_3_^2+^ but also enrich a large amount of TPrA from the solution via the hydrophobic interaction. Thus, using COF-LZU1 as an ECL microreactor, a confined microenvironment was well created for the redox and survival of coreactants. Through Nt. BbvCI restriction endonuclease-powered DNA walker and T7 exonuclease-mediated target recycling for dual signal amplification, these microreactors were further employed for ultrasensitive ECL detection of aflatoxin M1, showing a low LOD of 0.009 pg mL^−1^ [131].

**Fig. 10:**
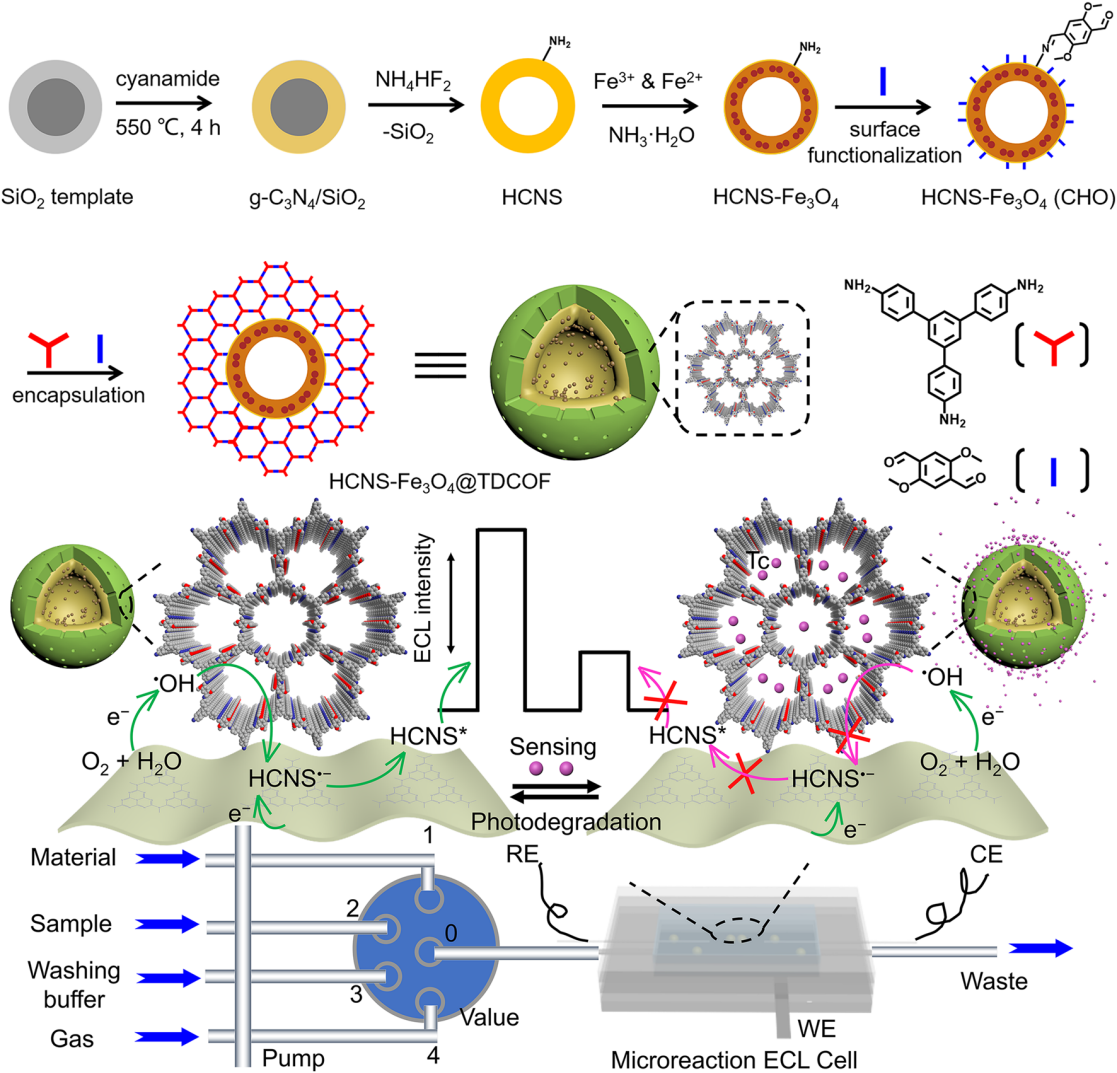
Schematic illustrations of the preparation progress of a biomimetic hollow organic crystalline nanosphere and the instrument and principle for Tc sensing. Reproduced with permission from Ref. [57]. Copyright (2022) Wiley–VCH

Microporous COFs can act as efficient adsorbents for small organic molecules due to the electrostatic, hydrophobic, hydrogen bonding, and *π*–*π* interactions [9, 57]. Thus, the presence of COFs can enrich target analytes and improve analytical behaviors. Our group designed a biomimetic hollow organic nanosphere by orderly hybridizing imine-linked COFs with hollow g-C_3_N_4_ nanosphere (Fig. 10). On the basis of the quenching effect of tetracycline (Tc) on the g-C_3_N_4_/O_2_ ECL system, these bioinspired nanospheres served as nanoprobes for ultrasensitive detection of Tc, and the LOD achieved a sub-part per billion level of 0.031 μg L^−1^. Experiments and theoretical derivation demonstrated that COF shells played an important role in enriching Tc, and the target amount is equivalent to being amplified around g-C_3_N_4_ accordingly. Besides, the Tc quencher and the coreactant radials traveled and mutually reacted within the narrow and long nanopore channels, ensuring again high quenching effect. Furthermore, a novel ECL technology utilizing the biomimetic self-responsiveness of these nanoprobes realized the evaluation of the photodegradation process [57].

In addition, COFs with high specific surface area and porosity can also serve as excellent electrode matrixes to support catalytic centers in a well-dispersed manner for amplified ECL outputs. For example, a liquid–liquid interface assembly strategy allowed for the polymerization of Zr-coordinated amide porphyrin-based COFs. This Zr-porphyrin-based COF adopted a 2D multilayer structure with high conductivity and electrocatalysis performance toward the luminol/H_2_O_2_ ECL system, which was further utilized to construct a novel MIP ECL sensor for Tc detection based on the gate control effect [149]. Li and co-workers exploited COF_TAPB-Dva_ as a carrier to load abundant Au nanoclusters for Pb^2+^ ECL sensing. In comparison with solid-state aggregated Au nanoclusters, the COF-confined ones displayed a 3.3-fold enhancement in anodic ECL efficiency. As such, the fabricated ECL biosensor obtained sensitive detection of Pb^2+^ down to 7.9 pM [150]. Ma et al. prepared a carbon dots (CDs)-based COF via the condensation reaction of aldehyde-CDs and TAPB, leading to the regular arrangement of CDs with efficient ECL emission in the presence of K_2_S_2_O_8_ and Bu_4_NPF_6_. Further combined with a CRISPR/Cas12a trans-cutting strategy, a high-performance ECL biosensor realized the BPA sensing with a LOD as low as 2.21 fM [151]. Besides, *N*-(4-aminobutyl)-*N*-ethylisoluminol (ABEI) was also linked to COF-LZU1 as an ECL emitter for label-free signal-off detection of cytochrome c. This sensor exhibited a wide linear range of 1.00 fg mL^−1^–0.10 ng mL^−1^ and a low LOD down to 0.73 fg mL^−1^ [152].

## Conclusions and Perspective

This review primarily provides an overview of the state-of-the-art COFs utilized in the field of electroanalytical chemistry, sketching the trajectory from basic characteristics and general synthesis strategies to the resultant sensing usages. The progress of elaborate COFs-based EC sensors is discussed systematically, including ions sensors, chemical sensors, immunosensors, and aptasensors. These COFs-based EC sensors demonstrated superior analytical performance. (1) Until now, imine-linked COFs formed by aminaldehyde condensation remain the most reported COFs for EC sensing applications due to their facile synthesis, high crystallinity, and stability. (2) Their high specific surface area provides a large electrode active area and high loading capacity, and the super-micropores and ordered channels ensure favorable energy/mass transfer. (3) Nano-sized pores and existing heteroatoms can trap guest targets through non-covalent interactions. (4) COFs generally exhibit flexibility, good biocompatibility, low toxicity, and high stability, beneficial for the fabrication of repeatable and reproducible EC sensors. (5) Framework networks designed by *π*-stacked or extended *π*-conjugated backbones, for example, that are COFs via pyrazine or olefin linkage, possess acceptable electrical conductivity. (6) Ratiometric EC sensing can be executed for accurate results based on the unique electroactive COFs.

In addition, ECL available COFs and their preliminary sensing applications are summarized in detail. (1) At present, modulating the IRCT behavior of COFs is a common strategy to activate ECL from non-ECL active monomers. (2) AIE-ECL COFs can be facilely achieved because the rigid framework restricts the intramolecular rotations and vibrations of luminogens. (3) Designing COFs with a fully *π*-conjugated planar structure can improve the conductivity and achieve fast charge injection. (4) The inherent characteristics of non-ECL active COFs make them the right microreactors, strong absorbents, and electrode matrices with high specific surface area, thereby improving the performance of ECL sensors.

Therefore, we believe that COFs are capable of taking a place in the field of electroanalytical chemistry, accompanied by the development of advanced synthetic methods and the disclosure of unique attributes in future work. Despite impressive progress, various obstacles remain to be addressed for the wide applications of COFs in electroanalytical chemistry. First, the solvothermal method still dominates the COFs preparation, but it usually requires tedious synthesis procedures and harsh reaction conditions. Second, COFs composed of non-conductive organic motifs are severely restricted as electroactive substances for EC sensing. The synthesis of highly conductive COFs is still relatively challenging work. Third, due to poor affinity, the assembly of COFs at the interface makes it difficult to achieve high efficiency and reproducibility. Most of the current works focus on the direct use of COFs while ignoring the vital role of modification strategies. Fourth, theoretical prediction of the covalent ordered structure of COFs and their EC behaviors is an arduous task. Lastly, the potential biotoxicity of COFs may affect their further commercial applications.

A few shallow outlooks are only for the reference of readers. In terms of the EC sensing aspect, it is a promising direction to develop conductive COFs with completely *π*-conjugated planar structures. Assembling COFs-derived composites with particular architecture can also access good conductivity and affinity. Usually, COFs prepared through the traditional solvothermal method exhibit nonuniform bulky morphology, so it is meaningful to develop synthetic strategies for special structures, such as hollow nanostructures mimicking containers, vesicles, and cells. Certainly, developing facile, green, and mild synthetic methods is a must for the large-scale manufacture of COFs-based EC sensors. Pre-functionalized monomers or post-synthetic modifications to exploit the diversity of species and functions of COFs have great potential for future development. Furthermore, most reported COFs possess only micropores, preventing guests (e.g., enzymes, quantum dots) from entering the active cavities or open channels, and creating hierarchical porosity in crystal COFs is highly desired accordingly. In particular, the *in-situ* interface assembly strategy of COFs awaits further exploration to establish stable and repeatable sensing platforms. In the ECL domain, although some ECL available COFs have been discovered, the ECL research on COFs is still in its initial stage. Future research is suggested to focus on the design of novel COFs emitters, the study of structure-related ECL behaviors, the dependence of their physicochemical properties and electronic structure on the ECL efficiency, the disclosure of underlying ECL reactions occurring in COFs, as well as expanding ECL assays.

## References

[1] A.P. Côté, A.I. Benin, N.W. Ockwig, M. O’Keeffe, A.J. Matzger et al., Porous, crystalline, covalent organic frameworks. Science **310**(5751), 1166–1170 (2005). 10.1126/science.1120411 16293756 10.1126/science.1120411

[2] K. Geng, T. He, R. Liu, S. Dalapati, K.T. Tan et al., Covalent organic frameworks: design, synthesis, and functions. Chem. Rev. **120**(16), 8814–8933 (2020). 10.1021/acs.chemrev.9b00550 31967791 10.1021/acs.chemrev.9b00550

[3] N. Huang, P. Wang, D. Jiang, Covalent organic frameworks: a materials platform for structural and functional designs. Nat. Rev. Mater. **1**(10), 16068 (2016). 10.1038/natrevmats.2016.68

[4] H. Xu, J. Gao, D. Jiang, Stable, crystalline, porous, covalent organic frameworks as a platform for chiral organocatalysts. Nat. Chem. **7**(11), 905–912 (2015). 10.1038/nchem.2352 26492011 10.1038/nchem.2352

[5] Y. Jin, Y. Hu, W. Zhang, Tessellated multiporous two-dimensional covalent organic frameworks. Nat. Rev. Chem. **1**(7), 0056 (2017). 10.1038/s41570-017-0056

[6] P. Pachfule, A. Acharjya, J. Roeser, T. Langenhahn, M. Schwarze et al., Diacetylene functionalized covalent organic framework (COF) for photocatalytic hydrogen generation. J. Am. Chem. Soc. **140**(4), 1423–1427 (2018). 10.1021/jacs.7b11255 29287143 10.1021/jacs.7b11255

[7] Y. Xiong, Q. Liao, Z. Huang, X. Huang, C. Ke et al., Ultrahigh responsivity photodetectors of 2D covalent organic frameworks integrated on graphene. Adv. Mater. **32**(9), 1907242 (2020). 10.1002/adma.201907242 10.1002/adma.201907242 31990415

[8] E. Vitaku, C.N. Gannett, K.L. Carpenter, L. Shen, H.D. Abruña et al., Phenazine-based covalent organic framework cathode materials with high energy and power densities. J. Am. Chem. Soc. **142**(1), 16–20 (2020). 10.1021/jacs.9b08147 31820958 10.1021/jacs.9b08147

[9] S. Karak, K. Dey, A. Torris, A. Halder, S. Bera et al., Inducing disorder in order: hierarchically porous covalent organic framework nanostructures for rapid removal of persistent organic pollutants. J. Am. Chem. Soc. **141**(18), 7572–7581 (2019). 10.1021/jacs.9b02706 31017396 10.1021/jacs.9b02706

[10] Q.Q. Jiang, Y.J. Li, Q. Wu, R.P. Liang, X. Wang et al., Molecular insertion: a master key to unlock smart photoelectric responses of covalent organic frameworks. Small **19**, e2302254 (2023). 10.1002/smll.202302254 37236205 10.1002/smll.202302254

[11] J. Chang, C. Li, X. Wang, D. Li, J. Zhang et al., Quasi-three-dimensional cyclotriphosphazene-based covalent organic framework nanosheet for efficient oxygen reduction. Nano-Micro Lett. **15**(1), 159 (2023). 10.1007/s40820-023-01111-8 10.1007/s40820-023-01111-8 PMC10310679 37386227

[12] E. Bakker, M. Telting-Diaz, Electrochemical sensors. Anal. Chem. **74**(12), 2781–2800 (2002). 10.1021/ac0202278 12090665 10.1021/ac0202278

[13] H. Liang, M. Xu, Y. Zhu, L. Wang, Y. Xie et al., H_2_O_2_ ratiometric electrochemical sensors based on nanospheres derived from ferrocence-modified covalent organic frameworks. ACS Appl. Nano Mater. **3**(1), 555–562 (2019). 10.1021/acsanm.9b02117

[14] H. Liang, L. Wang, Y. Yang, Y. Song, L. Wang, A novel biosensor based on multienzyme microcapsules constructed from covalent-organic framework. Biosens. Bioelectron. **193**, 113553 (2021). 10.1016/j.bios.2021.113553 34385018 10.1016/j.bios.2021.113553

[15] N. Karimian, P. Hashemi, A. Afkhami, H. Bagheri, The principles of bipolar electrochemistry and its electroanalysis applications. Curr. Opin. Electrochem. **17**, 30–37 (2019). 10.1016/j.coelec.2019.04.015

[16] A. Khanmohammadi, A. Jalili Ghazizadeh, P. Hashemi, A. Afkhami, F. Arduini et al., An overview to electrochemical biosensors and sensors for the detection of environmental contaminants. J. Iran. Chem. Soc. **17**(10), 2429–2447 (2020). 10.1007/s13738-020-01940-z

[17] Y. Cao, J.J. Zhu, Recent progress in electrochemiluminescence of halide perovskites. Front. Chem. **9**, 629830 (2021). 10.3389/fchem.2021.629830 33816436 10.3389/fchem.2021.629830 PMC8017205

[18] Y. Cao, Y. Zhou, Y. Lin, J.J. Zhu, Hierarchical metal-organic framework-confined CsPbBr_3_ quantum dots and aminated carbon dots: a new self-sustaining suprastructure for electrochemiluminescence bioanalysis. Anal. Chem. **93**(3), 1818–1825 (2021). 10.1021/acs.analchem.0c04717 33372764 10.1021/acs.analchem.0c04717

[19] T. Han, C. Ma, L. Wang, Y. Cao, H.Y. Chen et al., A novel electrochemiluminescence Janus emitter for dual-mode biosensing. Adv. Funct. Mater. **32**(24), 2200863 (2022). 10.1002/adfm.202200863

[20] Y. Cao, J.L. Zhou, Y. Ma, Y. Zhou, J.J. Zhu, Recent progress of metal nanoclusters in electrochemiluminescence. Dalton Trans. **51**(23), 8927–8937 (2022). 10.1039/d2dt00810f 35593102 10.1039/d2dt00810f

[21] C. Ma, Y. Cao, X. Gou, J.J. Zhu, Recent progress in electrochemiluminescence sensing and imaging. Anal. Chem. **92**(1), 431–454 (2020). 10.1021/acs.analchem.9b04947 31679341 10.1021/acs.analchem.9b04947

[22] C. Zhang, M. Cui, J. Ren, Y. Xing, N. Li et al., Facile synthesis of novel spherical covalent organic frameworks integrated with Pt nanoparticles and multiwalled carbon nanotubes as electrochemical probe for tanshinol drug detection. Chem. Eng. J. **401**, 126025 (2020). 10.1016/j.cej.2020.126025

[23] Y. Chen, Y. Xie, X. Sun, Y. Wang, Y. Wang, Tunable construction of crystalline and shape-tailored Co_3_O_4_@TAPB-DMTP-COF composites for the enhancement of tert-butylhydroquinone electrocatalysis. Sens. Actuators B Chem. **331**, 129438 (2021). 10.1016/j.snb.2021.129438

[24] J. Han, J. Yu, Y. Guo, L. Wang, Y. Song, COFBTLP^-1^/three-dimensional macroporous carbon electrode for simultaneous electrochemical detection o f Cd^2+^, Pb^2+^, Cu^2+^ and Hg^2+^. Sens. Actuators B Chem. **321**, 128498 (2020). 10.1016/j.snb.2020.128498

[25] X.Y. Ji, K. Sun, Z.K. Liu, X. Liu, W. Dong et al., Identification of dynamic active sites among Cu species derived from MOFs@CuPc for electrocatalytic nitrate reduction reaction to ammonia. Nano-Micro Lett. **15**(1), 110 (2023). 10.1007/s40820-023-01091-9 10.1007/s40820-023-01091-9 PMC10149566 37121962

[26] N. Karimian, P. Hashemi, A. Khanmohammadi, A. Afkhami, H. Bagheri, The principles and recent applications of bioelectrocatalysis. Anal. Bioanal. Chem. Res. **7**(3), 281–301 (2020). 10.22036/ABCR.2020.206676.1423

[27] M.M. Bordbar, A. Sheini, P. Hashemi, A. Hajian, H. Bagheri, Disposable paper-based biosensors for the point-of-care detection of hazardous contaminations-a review. Biosensors **11**(9), 316 (2021). 10.3390/bios11090316 34562906 10.3390/bios11090316 PMC8464915

[28] Y. Gao, J. Wang, Y. Yang, J. Wang, C. Zhang et al., Engineering spin states of isolated copper species in a metal–organic framework improves urea electrosynthesis. Nano-Micro Lett. **15**(1), 158 (2023). 10.1007/s40820-023-01127-0 10.1007/s40820-023-01127-0 PMC10284786 37341868

[29] H. Fakhri, H. Bagheri, Highly efficient Zr-MOF@WO_3_/graphene oxide photocatalyst: synthesis, characterization and photodegradation of tetracycline and malathion. Mat. Sci. Semicon. Proc. **107**, 104815 (2020). 10.1016/j.mssp.2019.104815

[30] N. Karimian, H. Fakhri, S. Amidi, A. Hajian, F. Arduini et al., A novel sensing layer based on metal-organic framework UiO-66 modified with TiO_2_-graphene oxide: application to rapid, sensitive and simultaneous determination of paraoxon and chlorpyrifos. New J. Chem. **43**(6), 2600–2609 (2019). 10.1039/c8nj06208k

[31] S.M. Khoshfetrat, H. Khoshsafar, A. Afkhami, M.A. Mehrgardi, H. Bagheri, Enhanced visual wireless electrochemiluminescence immunosensing of prostate-specific antigen based on the luminol loaded into MIL-53(Fe)-NH_2_ accelerator and hydrogen evolution reaction mediation. Anal. Chem. **91**(9), 6383–6390 (2019). 10.1021/acs.analchem.9b01506 30987423 10.1021/acs.analchem.9b01506

[32] H. Khoshsafar, N. Karimian, T.A. Nguyen, H. Fakhri, A. Khanmohammadi et al., Enzymeless voltammetric sensor for simultaneous determination of parathion and paraoxon based on Nd-based metal-organic framework. Chemosphere **292**, 133440 (2022). 10.1016/j.chemosphere.2021.133440 34973245 10.1016/j.chemosphere.2021.133440

[33] B. Cao, H. Liu, X. Zhang, P. Zhang, Q. Zhu et al., MOF-derived ZnS nanodots/Ti_3_C_2_Tx mxene hybrids boosting superior lithium storage performance. Nano-Micro Lett. **13**(1), 202 (2021). 10.1007/s40820-021-00728-x 10.1007/s40820-021-00728-x PMC8473522 34568995

[34] Z. Ye, Y. Jiang, L. Li, F. Wu, R. Chen, Rational design of mof-based materials for next-generation rechargeable batteries. Nano-Micro Lett. **13**(1), 203 (2021). 10.1007/s40820-021-00726-z 10.1007/s40820-021-00726-z PMC8492800 34611765

[35] X. Huang, J. Wei, Y. Zhang, B. Qian, Q. Jia et al., Ultralight magnetic and dielectric aerogels achieved by metal-organic framework initiated gelation of graphene oxide for enhanced microwave absorption. Nano-Micro Lett. **14**(1), 107 (2022). 10.1007/s40820-022-00851-3 10.1007/s40820-022-00851-3 PMC9019009 35438351

[36] G. Nagaraju, S.C. Sekhar, B. Ramulu, S.K. Hussain, D. Narsimulu et al., Ternary MOF-based redox active sites enabled 3D-on-2D nanoarchitectured battery-type electrodes for high-energy-density supercapatteries. Nano-Micro Lett. **13**(1), 17 (2020). 10.1007/s40820-020-00528-9 10.1007/s40820-020-00528-9 PMC8187485 34138181

[37] Z. Cao, R. Momen, S. Tao, D. Xiong, Z. Song et al., Metal-organic framework materials for electrochemical supercapacitors. Nano-Micro Lett. **14**(1), 181 (2022). 10.1007/s40820-022-00910-9 10.1007/s40820-022-00910-9 PMC9437182 36050520

[38] J. Chen, D. Sheng, T. Ying, H. Zhao, J. Zhang et al., MoFs-based nitric oxide therapy for tendon regeneration. Nano-Micro Lett. **13**(1), 23 (2020). 10.1007/s40820-020-00542-x 10.1007/s40820-020-00542-x PMC8187533 34138189

[39] X. He, Fundamental perspectives on the electrochemical water applications of metal-organic frameworks. Nano-Micro Lett. **15**(1), 148 (2023). 10.1007/s40820-023-01124-3 10.1007/s40820-023-01124-3 PMC10247659 37286907

[40] Y. Hu, H. Huang, D. Yu, X. Wang, L. Li et al., All-climate Aluminum-ion batteries based on binder-free mof-derived FeS_2_@C/CNT cathode. Nano-Micro Lett. **13**(1), 159 (2021). 10.1007/s40820-021-00682-8 10.1007/s40820-021-00682-8 PMC8302704 34297240

[41] J. Huang, P. Wu, Controlled assembly of luminescent lanthanide-organic frameworks via post-treatment of 3D-printed objects. Nano-Micro Lett. **13**(1), 15 (2020). 10.1007/s40820-020-00543-w 10.1007/s40820-020-00543-w PMC8187549 34138212

[42] Y. Lu, R. Zhou, N. Wang, Y. Yang, Z. Zheng et al., Engineer nanoscale defects into selective channels: MOF-enhanced Li^+^ separation by porous layered double hydroxide membrane. Nano-Micro Lett. **15**(1), 147 (2023). 10.1007/s40820-023-01101-w 10.1007/s40820-023-01101-w PMC10247908 37286909

[43] C. Li, Y. Ji, Y. Wang, C. Liu, Z. Chen et al., Applications of metal–organic frameworks and their derivatives in electrochemical CO_2_ reduction. Nano-Micro Lett. **15**(1), 113 (2023). 10.1007/s40820-023-01092-8 10.1007/s40820-023-01092-8 PMC10149437 37121938

[44] Y. Zhu, K. Yue, C. Xia, S. Zaman, H. Yang et al., Recent advances on MOF derivatives for non-noble metal oxygen electrocatalysts in zinc–air batteries. Nano-Micro Lett. **13**(1), 137 (2021). 10.1007/s40820-021-00669-5 10.1007/s40820-021-00669-5 PMC8184897 34138394

[45] X. Chen, L. Kong, J.A. Mehrez, C. Fan, W. Quan et al., Outstanding humidity chemiresistors based on imine-linked covalent organic framework films for human respiration monitoring. Nano-Micro Lett. **15**(1), 149 (2023). 10.1007/s40820-023-01107-4 10.1007/s40820-023-01107-4 PMC10247948 37286913

[46] R. Yuan, H.K. Li, H. He, Recent advances in metal/covalent organic framework-based electrochemical aptasensors for biosensing applications. Dalton Trans. **50**(40), 14091–14104 (2021). 10.1039/d1dt02360h 34609402 10.1039/d1dt02360h

[47] X. Zhao, P. Pachfule, A. Thomas, Covalent organic frameworks (COFs) for electrochemical applications. Chem. Soc. Rev. **50**(12), 6871–6913 (2021). 10.1039/d0cs01569e 33881422 10.1039/d0cs01569e

[48] X. Zhang, G. Li, D. Wu, B. Zhang, N. Hu et al., Recent advances in the construction of functionalized covalent organic frameworks and their applications to sensing. Biosens. Bioelectron. **145**, 111699 (2019). 10.1016/j.bios.2019.111699 31563802 10.1016/j.bios.2019.111699

[49] B. Mohan, R. Kumari, Virender, G. Singh, K. Singh et al., Covalent organic frameworks (COFs) and metal–organic frameworks (MOFs) as electrochemical sensors for the efficient detection of pharmaceutical residues. Environ. Int. **175**, 107928 (2023). 10.1016/j.envint.2023.107928 37094512 10.1016/j.envint.2023.107928

[50] X. Xu, Z. Zhang, R. Xiong, G. Lu, J. Zhang et al., Bending resistance covalent organic framework superlattice: “nano-hourglass”-induced charge accumulation for flexible in-plane micro-supercapacitors. Nano-Micro Lett. **15**(1), 25 (2022). 10.1007/s40820-022-00997-0 10.1007/s40820-022-00997-0 PMC9803805 36583830

[51] G. Yan, X. Sun, Y. Zhang, H. Li, H. Huang et al., Metal-free 2D/2D van der waals heterojunction based on covalent organic frameworks for highly efficient solar energy catalysis. Nano-Micro Lett. **15**(1), 132 (2023). 10.1007/s40820-023-01100-x 10.1007/s40820-023-01100-x PMC10200743 37211571

[52] T. Zhang, C. Gao, W. Huang, Y. Chen, Y. Wang et al., Covalent organic framework as a novel electrochemical platform for highly sensitive and stable detection of lead. Talanta **188**, 578–583 (2018). 10.1016/j.talanta.2018.06.032 30029415 10.1016/j.talanta.2018.06.032

[53] F. Pan, C. Tong, Z. Wang, H. Han, P. Liu et al., Nanocomposite based on graphene and intercalated covalent organic frameworks with hydrosulphonyl groups for electrochemical determination of heavy metal ions. Microchim. Acta **188**(9), 295 (2021). 10.1007/s00604-021-04956-1 10.1007/s00604-021-04956-1 34379203

[54] H. Wang, J. Zhao, Y. Li, Y. Cao, Z. Zhu et al., Aqueous two-phase interfacial assembly of COF membranes for water desalination. Nano-Micro Lett. **14**(1), 216 (2022). 10.1007/s40820-022-00968-5 10.1007/s40820-022-00968-5 PMC9646690 36352333

[55] O. Yildirim, B. Derkus, Triazine-based 2D covalent organic frameworks improve the electrochemical performance of enzymatic biosensors. J. Mater. Sci. **55**(7), 3034–3044 (2019). 10.1007/s10853-019-04254-5

[56] X. Liu, M. Hu, M. Wang, Y. Song, N. Zhou et al., Novel nanoarchitecture of Co-MOF-on-TPN-COF hybrid: ultralowly sensitive bioplatform of electrochemical aptasensor toward ampicillin. Biosens. Bioelectron. **123**, 59–68 (2019). 10.1016/j.bios.2018.09.089 30312876 10.1016/j.bios.2018.09.089

[57] Y. Cao, R. Wu, Y. Zhou, D. Jiang, W. Zhu, A bioinspired photocatalysis and electrochemiluminescence scaffold for simultaneous degradation and in situ evaluation. Adv. Funct. Mater. **32**(31), 2203005 (2022). 10.1002/adfm.202203005

[58] J.L. Zhang, L.Y. Yao, Y. Yang, W.B. Liang, R. Yuan et al., Conductive covalent organic frameworks with conductivity- and pre-reduction-enhanced electrochemiluminescence for ultrasensitive biosensor construction. Anal. Chem. **94**(8), 3685–3692 (2022). 10.1021/acs.analchem.1c05436 35156809 10.1021/acs.analchem.1c05436

[59] Y.J. Li, W.R. Cui, Q.Q. Jiang, Q. Wu, R.P. Liang et al., A general design approach toward covalent organic frameworks for highly efficient electrochemiluminescence. Nat. Commun. **12**(1), 4735 (2021). 10.1038/s41467-021-25013-8 34354067 10.1038/s41467-021-25013-8 PMC8342611

[60] Y. Sun, G.I.N. Waterhouse, L. Xu, X. Qiao, Z. Xu, Three-dimensional electrochemical sensor with covalent organic framework decorated carbon nanotubes signal amplification for the detection of furazolidone. Sens. Actuators B Chem. **321**, 128501 (2020). 10.1016/j.snb.2020.128501

[61] L. Wang, Y. Song, Y. Luo, L. Wang, A novel covalent organic framework with multiple adsorption sites for removal of Hg^2+^ and sensitive detection of nitrofural. J. Ind. Eng. Chem. **106**, 374–381 (2022). 10.1016/j.jiec.2021.11.014

[62] D. Li, H. Zhao, G. Wang, R. Liu, L. Bai, Room-temperature ultrasonic-assisted self-assembled synthesis of silkworm cocoon-like COFs@GCNTs composite for sensitive detection of diuron in food samples. Food Chem. **418**, 135999 (2023). 10.1016/j.foodchem.2023.135999 37001360 10.1016/j.foodchem.2023.135999

[63] B. Liu, H. Guo, L. Sun, Z. Pan, L. Peng et al., Electrochemical sensor based on covalent organic frameworks/MWCNT for simultaneous detection of catechol and hydroquinone. Colloids Surf. A **639**, 128335 (2022). 10.1016/j.colsurfa.2022.128335

[64] H. Guo, B. Liu, Z. Pan, L. Sun, L. Peng et al., Electrochemical determination of dopamine and uric acid with covalent organic frameworks and Ox-MWCNT co-modified glassy carbon electrode. Colloids Surf. A **648**, 129316 (2022). 10.1016/j.colsurfa.2022.129316

[65] Z. Pan, Y. Wei, H. Guo, B. Liu, L. Sun et al., Sensitive detection of sulfamethoxazole by an electrochemical sensing platform with a covalent organic framework in situ grown on polyaniline. Microporous Mesoporous Mater. **348**, 112409 (2023). 10.1016/j.micromeso.2022.112409

[66] S. Lu, S. Wang, P. Wu, D. Wang, J. Yi et al., A composite prepared from covalent organic framework and gold nanoparticles for the electrochemical determination of enrofloxacin. Adv. Powder Technol. **32**(6), 2106–2115 (2021). 10.1016/j.apt.2021.04.025

[67] T. Zhang, Y. Chen, W. Huang, Y. Wang, X. Hu, A novel AuNPs-doped COFs composite as electrochemical probe for chlorogenic acid detection with enhanced sensitivity and stability. Sens. Actuators B Chem. **276**, 362–369 (2018). 10.1016/j.snb.2018.08.132

[68] Q. Guan, H. Guo, R. Xue, M. Wang, N. Wu et al., Electrochemical sensing platform based on covalent organic framework materials and gold nanoparticles for high sensitivity determination of theophylline and caffeine. Microchim. Acta **188**(3), 85 (2021). 10.1007/s00604-021-04744-x 10.1007/s00604-021-04744-x 33587169

[69] X. Zhang, J. Zhu, Z. Wu, W. Wen, X. Zhang et al., Electrochemical sensor based on confined synthesis of gold nanoparticles@covalent organic frameworks for the detection of bisphenol A. Anal. Chim. Acta **1239**, 340743 (2023). 10.1016/j.aca.2022.340743 36628736 10.1016/j.aca.2022.340743

[70] H. Zhao, K. Shi, C. Zhang, J. Ren, M. Cui et al., Spherical COFs decorated with gold nanoparticles and multiwalled carbon nanotubes as signal amplifier for sensitive electrochemical detection of doxorubicin. Microchem. J. **182**, 107865 (2022). 10.1016/j.microc.2022.107865

[71] R. Chen, X. Peng, Y. Song, Y. Du, A paper-based electrochemical sensor based on PtNP/COFTFPB-DHzDS@rGO for sensitive detection of furazolidone. Biosensors **12**(10), 904 (2022). 10.3390/bios12100904 36291041 10.3390/bios12100904 PMC9599777

[72] Q. Guan, H. Guo, R. Xue, M. Wang, X. Zhao et al., Electrochemical sensor based on covalent organic frameworks-MWCNT-NH_2_/AuNPs for simultaneous detection of dopamine and uric acid. J. Electroanal. Chem. **880**, 114932 (2021). 10.1016/j.jelechem.2020.114932

[73] Z. Pan, H. Guo, B. Liu, L. Sun, Y. Chen et al., A sensitive electrochemical sensing platform based on nitrogen-rich covalent organic framework for simultaneous detection of guanine and adenine. Microporous Mesoporous Mater. **340**, 112030 (2022). 10.1016/j.micromeso.2022.112030

[74] W. Wei, S. Zhou, D.D. Ma, Q. Li, M. Ran et al., Ultrathin conductive bithiazole-based covalent organic framework nanosheets for highly efficient electrochemical biosensing. Adv. Funct. Mater. **33**(36), 2302917 (2023). 10.1002/adfm.202302917

[75] X. Lin, Y. Deng, Y. He, J. Chen, S. Hu, Construction of hydrophilic N, O-rich carboxylated triazine-covalent organic frameworks for the application in selective simultaneous electrochemical detection. Appl. Surf. Sci. **545**, 149047 (2021). 10.1016/j.apsusc.2021.149047

[76] X. Tan, Y. Fan, S. Wang, Y. Wu, W. Shi, Ultrasensitive and highly selective electrochemical sensing of sodium picrate by dihydroxylatopillar[6]arene-modified gold nanoparticles and cationic pillar[6]arene functionalized covalent organic framework. Electrochim. Acta **335**, 135706 (2020). 10.1016/j.electacta.2020.135706

[77] Y. Xiao, N. Wu, L. Wang, L. Chen, A novel paper-based electrochemical biosensor based on N, O-rich covalent organic frameworks for carbaryl detection. Biosensors **12**(10), 899 (2022). 10.3390/bios12100899 36291036 10.3390/bios12100899 PMC9599374

[78] L. Wang, H. Liang, M. Xu, L. Wang, Y. Xie et al., Ratiometric electrochemical biosensing based on double-enzymes loaded on two-dimensional dual-pore COFETTA-TPAL. Sens. Actuators B Chem. **298**, 126859 (2019). 10.1016/j.snb.2019.126859

[79] X. Zha, X. Sun, H. Chu, Y. Wang, Synthesis of bimetallic covalent organic framework nanocomposite for enhanced electrochemical detection of gallic acid. Colloids Surf. A **651**, 129748 (2022). 10.1016/j.colsurfa.2022.129748

[80] H. Chu, X. Sun, X. Zha, Y. Zhang, Y. Wang, Synthesis of core-shell structured metal oxide@covalent organic framework composites as a novel electrochemical platform for dopamine sensing. Colloids Surf. A **648**, 129238 (2022). 10.1016/j.colsurfa.2022.129238

[81] Y. Xie, M. Xu, L. Wang, H. Liang, L. Wang et al., Iron-porphyrin-based covalent-organic frameworks for electrochemical sensing H_2_O_2_ and pH. Mater. Sci. Eng. C **112**, 110864 (2020). 10.1016/j.msec.2020.110864 10.1016/j.msec.2020.110864 32409033

[82] C. Zhang, L. Fan, J. Ren, M. Cui, N. Li et al., Facile synthesis of surface functionalized Pd^2+^@P-CDP/COFs for highly sensitive detection of norfloxacin drug based on the host-guest interaction. J. Pharm. Biomed. Anal. **219**, 114956 (2022). 10.1016/j.jpba.2022.114956 35882178 10.1016/j.jpba.2022.114956

[83] L. Wang, Y. Xie, Y. Yang, H. Liang, L. Wang et al., Electroactive covalent organic frameworks/carbon nanotubes composites for electrochemical sensing. ACS Appl. Nano Mater. **3**(2), 1412–1419 (2020). 10.1021/acsanm.9b02257

[84] N. Wu, L. Wang, Y. Xie, Y. Du, Y. Song et al., Double signal ratiometric electrochemical riboflavin sensor based on macroporous carbon/electroactive thionine-contained covalent organic framework. J. Colloids Interface Sci. **608**(1), 219–226 (2022). 10.1016/j.jcis.2021.09.162 10.1016/j.jcis.2021.09.162 34626968

[85] M. Xu, L. Wang, Y. Xie, Y. Song, L. Wang, Ratiometric electrochemical sensing and biosensing based on multiple redox-active state COFDHTA-TTA. Sens. Actuators B Chem. **281**, 1009–1015 (2019). 10.1016/j.snb.2018.11.032

[86] Y.H. Pang, Y.Y. Wang, X.F. Shen, J.Y. Qiao, Covalent organic framework modified carbon cloth for ratiometric electrochemical sensing of bisphenol A and S. Microchim. Acta **189**(5), 189 (2022). 10.1007/s00604-022-05297-3 10.1007/s00604-022-05297-3 35412090

[87] M.R. Jalali Sarvestani, T. Madrakian, A. Afkhami, Ultra-trace levels voltammetric determination of Pb^2+^ in the presence of Bi^3+^ at food samples by a Fe_3_O_4_@schiff base network1 modified glassy carbon electrode. Talanta **250**, 123716 (2022). 10.1016/j.talanta.2022.123716 35792444 10.1016/j.talanta.2022.123716

[88] L. Wang, Y. Yang, H. Liang, N. Wu, X. Peng et al., A novel N, S-rich COF and its derived hollow N, S-doped carbon@Pd nanorods for electrochemical detection of Hg^2+^ and paracetamol. J. Hazard. Mater. **409**, 124528 (2021). 10.1016/j.jhazmat.2020.124528 33234399 10.1016/j.jhazmat.2020.124528

[89] L. Pei, J. Su, H. Yang, Y. Wu, Y. Du et al., A novel covalent-organic framework for highly sensitive detection of Cd^2+^, Pb^2+^, Cu^2+^ and Hg^2+^. Microporous Mesoporous Mater. **333**, 111742 (2022). 10.1016/j.micromeso.2022.111742

[90] J. Han, L. Pei, Y. Du, Y. Zhu, Tripolycyanamide-2,4,6-triformyl pyrogallol covalent organic frameworks with many coordination sites for detection and removal of heavy metal ions. J. Ind. Eng. Chem. **107**, 53–60 (2022). 10.1016/j.jiec.2021.11.027

[91] L. Yu, L. Sun, Q. Zhang, J. Zhang, B. Yang et al., Highly efficient determination of Mn^2+^ in chinese liquor by using a novel electrochemical sensor based on TiO_2_-NH_2_@covalent organic framework nanocomposites. Anal. Methods **15**(21), 2622–2630 (2023). 10.1039/d3ay00222e 37194496 10.1039/d3ay00222e

[92] S. Feng, M. Yan, Y. Xue, J. Huang, X. Yang, Electrochemical immunosensor for cardiac troponin I detection based on covalent organic framework and enzyme-catalyzed signal amplification. Anal. Chem. **93**(40), 13572–13579 (2021). 10.1021/acs.analchem.1c02636 34591449 10.1021/acs.analchem.1c02636

[93] T. Zhang, N. Ma, A. Ali, Q. Wei, D. Wu et al., Electrochemical ultrasensitive detection of cardiac troponin I using covalent organic frameworks for signal amplification. Biosens. Bioelectron. **119**, 176–181 (2018). 10.1016/j.bios.2018.08.020 30125879 10.1016/j.bios.2018.08.020

[94] H. Liang, H. Xu, Y. Zhao, J. Zheng, H. Zhao et al., Ultrasensitive electrochemical sensor for prostate specific antigen detection with a phosphorene platform and magnetic covalent organic framework signal amplifier. Biosens. Bioelectron. **144**, 111691 (2019). 10.1016/j.bios.2019.111691 31520964 10.1016/j.bios.2019.111691

[95] T.Z. Liu, R. Hu, X. Zhang, K.L. Zhang, Y. Liu et al., Metal-organic framework nanomaterials as novel signal probes for electron transfer mediated ultrasensitive electrochemical immunoassay. Anal. Chem. **88**(24), 12516–12523 (2016). 10.1021/acs.analchem.6b04191 28193012 10.1021/acs.analchem.6b04191

[96] H. Boyacıoğlu, B.B. Yola, C. Karaman, O. Karaman, N. Atar et al., A novel electrochemical kidney injury molecule-1 (KIM-1) immunosensor based covalent organic frameworks-gold nanoparticles composite and porous NiCo_2_S_4_@CeO_2_ microspheres: the monitoring of acute kidney injury. Appl. Surf. Sci. **578**, 152093 (2022). 10.1016/j.apsusc.2021.152093

[97] Y. Chen, S. Wang, J. Ren, H. Zhao, M. Cui et al., Electrocatalysis of copper sulfide nanoparticle-engineered covalent organic frameworks for ratiometric electrochemical detection of amyloid-beta oligomer. Anal. Chem. **94**(32), 11201–11208 (2022). 10.1021/acs.analchem.2c01602 35920591 10.1021/acs.analchem.2c01602

[98] H. Liu, S. Ma, G. Ning, R. Zhang, H. Liang et al., A “peptide-target-aptamer” electrochemical biosensor for norovirus detection using a black phosphorous nanosheet@Ti_3_C_2_-Mxene nanohybrid and magnetic covalent organic framework. Talanta **258**, 124433 (2023). 10.1016/j.talanta.2023.124433 36996585 10.1016/j.talanta.2023.124433

[99] Z. Chen, M. Yang, Z. Li, W. Liao, B. Chen et al., Highly sensitive and convenient aptasensor based on Au NPs@Ce-TpBpy COF for quantitative determination of zearalenone. RSC Adv. **12**(27), 17312–17320 (2022). 10.1039/d2ra02093a 35765447 10.1039/d2ra02093a PMC9192137

[100] Z. Song, J. Song, F. Gao, X. Chen, Q. Wang et al., Novel electroactive ferrocene-based covalent organic frameworks towards electrochemical label-free aptasensors for the detection of cardiac troponin i. Sens. Actuators B Chem. **368**, 132205 (2022). 10.1016/j.snb.2022.132205

[101] Q.Q. Zhu, W.W. Zhang, H.W. Zhang, R. Yuan, H. He, Elaborately manufacturing an electrochemical aptasensor based on gold nanoparticle/COF composites for amplified detection performance. J. Mater. Chem. C **8**(47), 16984–16991 (2020). 10.1039/d0tc04202a

[102] Q.Q. Zhu, H.K. Li, X.L. Sun, Z.Y. Han, J. Sun et al., Rational incorporation of covalent organic framework/carbon nanotube (COF/CNT) composites for electrochemical aptasensing of ultra-trace atrazine. J. Mater. Chem. C **9**(25), 8043–8050 (2021). 10.1039/d1tc01506k

[103] M. Wang, M. Hu, J. Liu, C. Guo, D. Peng et al., Covalent organic framework-based electrochemical aptasensors for the ultrasensitive detection of antibiotics. Biosens. Bioelectron. **132**, 8–16 (2019). 10.1016/j.bios.2019.02.040 30851495 10.1016/j.bios.2019.02.040

[104] X. Yan, Y. Song, J. Liu, N. Zhou, C. Zhang et al., Two-dimensional porphyrin-based covalent organic framework: a novel platform for sensitive epidermal growth factor receptor and living cancer cell detection. Biosens. Bioelectron. **126**, 734–742 (2019). 10.1016/j.bios.2018.11.047 30553103 10.1016/j.bios.2018.11.047

[105] Z. He, J. Goulas, E. Parker, Y. Sun, X.D. Zhou et al., Review on covalent organic frameworks and derivatives for electrochemical and photocatalytic CO_2_ reduction. Catal. Today **409**, 103–118 (2023). 10.1016/j.cattod.2022.04.021

[106] T. Zhang, Y. Song, Y. Xing, Y. Gu, X. Yan et al., The synergistic effect of Au-COF nanosheets and artificial peroxidase Au@ZIF-8(NiPd) rhombic dodecahedra for signal amplification for biomarker detection. Nanoscale **11**(42), 20221–20227 (2019). 10.1039/c9nr07190c 31621739 10.1039/c9nr07190c

[107] Y. Chen, W. Li, X.H. Wang, R.Z. Gao, A.N. Tang et al., Green synthesis of covalent organic frameworks based on reaction media. Mater. Chem. Front. **5**(3), 1253–1267 (2021). 10.1039/d0qm00801j

[108] H. Wang, Z. Zeng, P. Xu, L. Li, G. Zeng et al., Recent progress in covalent organic framework thin films: fabrications, applications and perspectives. Chem. Soc. Rev. **48**(2), 488–516 (2019). 10.1039/c8cs00376a 30565610 10.1039/c8cs00376a

[109] X. Huang, L. Li, S. Zhao, L. Tong, Z. Li et al., MOF-like 3D graphene-based catalytic membrane fabricated by one-step laser scribing for robust water purification and green energy production. Nano-Micro Lett. **14**(1), 174 (2022). 10.1007/s40820-022-00923-4 10.1007/s40820-022-00923-4 PMC9399326 35999381

[110] J. Qiao, X. Zhang, C. Liu, L. Lyu, Y. Yang et al., Non-magnetic bimetallic MOF-derived porous carbon-wrapped TiO_2_/ZrTiO_4_ composites for efficient electromagnetic wave absorption. Nano-Micro Lett. **13**(1), 75 (2021). 10.1007/s40820-021-00606-6 10.1007/s40820-021-00606-6 PMC8187513 34138308

[111] C. Qiu, K. Qian, J. Yu, M. Sun, S. Cao et al., MOF-transformed In_2_O_3-x_@C nanocorn electrocatalyst for efficient CO_2_ reduction to HCOOH. Nano-Micro Lett. **14**(1), 167 (2022). 10.1007/s40820-022-00913-6 10.1007/s40820-022-00913-6 PMC9385936 35976472

[112] V. Schroeder, S. Savagatrup, M. He, S. Lin, T.M. Swager, Carbon nanotube chemical sensors. Chem. Rev. **119**(1), 599–663 (2019). 10.1021/acs.chemrev.8b00340 30226055 10.1021/acs.chemrev.8b00340 PMC6399066

[113] Z. Wang, L. Dong, W. Huang, H. Jia, Q. Zhao et al., Simultaneously regulating uniform Zn^2+^ flux and electron conduction by mof/rgo interlayers for high-performance Zn anodes. Nano-Micro Lett. **13**(1), 73 (2021). 10.1007/s40820-021-00594-7 10.1007/s40820-021-00594-7 PMC8187534 34138302

[114] H. Chu, X. Sun, X. Zha, S.U. Khan, Y. Wang, Ultrasensitive electrochemical detection of butylated hydroxy anisole via metalloporphyrin covalent organic frameworks possessing variable catalytic active sites. Biosensors **12**(11), 975 (2022). 10.3390/bios12110975 36354484 10.3390/bios12110975 PMC9688419

[115] T. Yang, R. Yu, Y. Yan, H. Zeng, S. Luo et al., A review of ratiometric electrochemical sensors: from design schemes to future prospects. Sens. Actuators B Chem. **274**, 501–516 (2018). 10.1016/j.snb.2018.07.138

[116] Y. Cao, C. Ma, J.J. Zhu, DNA technology-assisted signal amplification strategies in electrochemiluminescence bioanalysis. J. Anal. Test. **5**(2), 95–111 (2021). 10.1007/s41664-021-00175-y

[117] J. Cao, P. Ouyang, S. Yu, F. Shi, C. Ren et al., Hedgehog-like Bi2S3 nanostructures: a novel composite soft template route to the synthesis and sensitive electrochemical immunoassay of the liver cancer biomarker. Chem. Commun. **57**(14), 1766–1769 (2021). 10.1039/d0cc07572h 10.1039/d0cc07572h 33470257

[118] T. Han, Y. Cao, H.Y. Chen, J.J. Zhu, Versatile porous nanomaterials for electrochemiluminescence biosensing: recent advances and future perspective. J. Electroanal. Chem. **902**, 115821 (2021). 10.1016/j.jelechem.2021.115821

[119] Y. Zhang, B.S. Lai, M. Juhas, Recent advances in aptamer discovery and applications. Molecules **24**(5), 941 (2019). 10.3390/molecules24050941 30866536 10.3390/molecules24050941 PMC6429292

[120] Y.W. Zhang, Y. Cao, C.J. Mao, D. Jiang, W. Zhu, An iron(III)-based metal-organic gel-catalyzed dual electrochemiluminescence system for cytosensing and in situ evaluation of the VEGF165 subtype. Anal. Chem. **94**(9), 4095–4102 (2022). 10.1021/acs.analchem.2c00032 35196001 10.1021/acs.analchem.2c00032

[121] S.M. Khoshfetrat, K. Fasihi, F. Moradnia, H. Kamil Zaidan, E. Sanchooli, A label-free multicolor colorimetric and fluorescence dual mode biosensing of HIV-1 DNA based on the bifunctional NiFe_2_O_4_@UiO-66 nanozyme. Anal. Chim. Acta **1252**, 341073 (2023). 10.1016/j.aca.2023.341073 36935160 10.1016/j.aca.2023.341073

[122] S.M. Khoshfetrat, P.S. Dorraji, L. Fotouhi, M. Hosseini, F. Khatami et al., Enhanced electrochemiluminescence biosensing of gene-specific methylation in thyroid cancer patients’ plasma based integrated graphitic carbon nitride-encapsulated metal-organic framework nanozyme optimized by central composite design. Sens. Actuators B Chem. **364**, 131895 (2022). 10.1016/j.snb.2022.131895

[123] S.M. Khoshfetrat, P. Seyed Dorraji, M. Shayan, F. Khatami, K. Omidfar, Smartphone-based electrochemiluminescence for visual simultaneous detection of RASSF1A and SLC5A8 tumor suppressor gene methylation in thyroid cancer patient plasma. Anal. Chem. **94**(22), 8005–8013 (2022). 10.1021/acs.analchem.2c01132 35616262 10.1021/acs.analchem.2c01132

[124] A. Ahmadi, S.M. Khoshfetrat, Z. Mirzaeizadeh, S. Kabiri, J. Rezaie et al., Electrochemical immunosensor for determination of cardiac troponin i using two-dimensional metal-organic framework/Fe_3_O_4_–COOH nanosheet composites loaded with thionine and pCTAB/DES modified electrode. Talanta **237**, 122911 (2022). 10.1016/j.talanta.2021.122911 34736648 10.1016/j.talanta.2021.122911

[125] S.M. Khoshfetrat, P. Hashemi, A. Afkhami, A. Hajian, H. Bagheri, Cascade electrochemiluminescence-based integrated graphitic carbon nitride-encapsulated metal-organic framework nanozyme for prostate-specific antigen biosensing. Sens. Actuators B Chem. **348**, 130658 (2021). 10.1016/j.snb.2021.130658

[126] H. Zhao, F. Wang, L. Cui, X. Xu, X. Han et al., Composition optimization and microstructure design in mofs-derived magnetic carbon-based microwave absorbers: a review. Nano-Micro Lett. **13**(1), 208 (2021). 10.1007/s40820-021-00734-z 10.1007/s40820-021-00734-z PMC8505592 34633562

[127] Z. Zhang, Z. Cai, Z. Wang, Y. Peng, L. Xia et al., A review on metal-organic framework-derived porous carbon-based novel microwave absorption materials. Nano-Micro Lett. **13**(1), 56 (2021). 10.1007/s40820-020-00582-3 10.1007/s40820-020-00582-3 PMC8187524 34138258

[128] X. Zhang, J. Qiao, Y. Jiang, F. Wang, X. Tian et al., Carbon-based MOF derivatives: emerging efficient electromagnetic wave absorption agents. Nano-Micro Lett. **13**(1), 135 (2021). 10.1007/s40820-021-00658-8 10.1007/s40820-021-00658-8 PMC8180543 34138364

[129] R. Luo, H. Lv, Q. Liao, N. Wang, J. Yang et al., Intrareticular charge transfer regulated electrochemiluminescence of donor-acceptor covalent organic frameworks. Nat. Commun. **12**(1), 6808 (2021). 10.1038/s41467-021-27127-5 34815403 10.1038/s41467-021-27127-5 PMC8611053

[130] W.R. Cui, Y.J. Li, Q.Q. Jiang, Q. Wu, R.P. Liang et al., Tunable covalent organic framework electrochemiluminescence from non-electroluminescent monomers. Cell Rep. Phys. Sci. **3**(2), 100630 (2022). 10.1016/j.xcrp.2021.100630

[131] W.J. Zeng, K. Wang, W.B. Liang, Y.Q. Chai, R. Yuan et al., Covalent organic frameworks as micro-reactors: confinement-enhanced electrochemiluminescence. Chem. Sci. **11**(21), 5410–5414 (2020). 10.1039/d0sc01817a 34094067 10.1039/d0sc01817a PMC8159293

[132] Y. Cao, W. Zhu, H. Wei, C. Ma, Y. Lin et al., Stable and monochromatic all-inorganic halide perovskite assisted by hollow carbon nitride nanosphere for ratiometric electrochemiluminescence bioanalysis. Anal. Chem. **92**(5), 4123–4130 (2020). 10.1021/acs.analchem.0c00070 32046479 10.1021/acs.analchem.0c00070

[133] Y.J. Li, W.R. Cui, Q.Q. Jiang, R.P. Liang, X.J. Li et al., Arousing electrochemiluminescence out of non-electroluminescent monomers within covalent organic frameworks. ACS Appl. Mater. Interfaces **13**(40), 47921–47931 (2021). 10.1021/acsami.1c12958 34601862 10.1021/acsami.1c12958

[134] W.R. Cui, Y.J. Li, Q.Q. Jiang, Q. Wu, Q.X. Luo et al., Covalent organic frameworks as advanced uranyl electrochemiluminescence monitoring platforms. Anal. Chem. **93**(48), 16149–16157 (2021). 10.1021/acs.analchem.1c03907 34792351 10.1021/acs.analchem.1c03907

[135] S. Carrara, A. Aliprandi, C.F. Hogan, L. De Cola, Aggregation-induced electrochemiluminescence of platinum(II) complexes. J. Am. Chem. Soc. **139**(41), 14605–14610 (2017). 10.1021/jacs.7b07710 28914532 10.1021/jacs.7b07710

[136] X. Wei, M.J. Zhu, Z. Cheng, M. Lee, H. Yan et al., Aggregation-induced electrochemiluminescence of carboranyl carbazoles in aqueous media. Angew. Chem. Int. Ed. **58**(10), 3162–3166 (2019). 10.1002/anie.201900283 10.1002/anie.201900283 30698911

[137] T. Han, Y. Cao, J. Wang, J. Jiao, Y. Song et al., Crystallization-induced enhanced electrochemiluminescence from a new tris(bipyridine)ruthenium(II) derivative. Adv. Funct. Mater. **33**(12), 2212394 (2023). 10.1002/adfm.202212394

[138] Q.X. Luo, W.R. Cui, Y.J. Li, Y.J. Cai, X.L. Mao et al., Construction of sp^2^ carbon-conjugated covalent organic frameworks for framework-induced electrochemiluminescence. ACS Appl. Electron. Mater. **3**(10), 4490–4497 (2021). 10.1021/acsaelm.1c00636

[139] S. Li, X. Ma, C. Pang, M. Wang, G. Yin et al., Novel chloramphenicol sensor based on aggregation-induced electrochemiluminescence and nanozyme amplification. Biosens. Bioelectron. **176**, 112944 (2021). 10.1016/j.bios.2020.112944 33421761 10.1016/j.bios.2020.112944

[140] S. Li, C. Pang, X. Ma, Y. Wu, M. Wang et al., Aggregation-induced electrochemiluminescence and molecularly imprinted polymer based sensor with Fe_3_O_4_@Pt nanoparticle amplification for ultrasensitive ciprofloxacin detection. Microchem. J. **178**, 107345 (2022). 10.1016/j.microc.2022.107345

[141] J.L. Zhang, Y. Yang, W.B. Liang, L.Y. Yao, R. Yuan et al., Highly stable covalent organic framework nanosheets as a new generation of electrochemiluminescence emitters for ultrasensitive microrna detection. Anal. Chem. **93**(6), 3258–3265 (2021). 10.1021/acs.analchem.0c04931 33529534 10.1021/acs.analchem.0c04931

[142] L. Cui, C.Y. Zhu, J. Hu, X.M. Meng, M. Jiang et al., Construction of a dual-mode biosensor for electrochemiluminescent and electrochemical sensing of alkaline phosphatase. Sens. Actuators B Chem. **374**, 132779 (2023). 10.1016/j.snb.2022.132779

[143] Y. Yang, H. Jiang, J. Li, J. Zhang, S.Z. Gao et al., Highly stable Ru-complex-based metal-covalent organic frameworks as novel type of electrochemiluminescence emitters for ultrasensitive biosensing. Mater. Horiz. **10**, 3005–3013 (2023). 10.1039/d3mh00260h 37194328 10.1039/d3mh00260h

[144] L. Song, W. Gao, S. Wang, H. Bi, S. Deng et al., Construction of an aminal-linked covalent organic framework-based electrochemiluminescent sensor for enantioselective sensing phenylalanine. Sens. Actuators B Chem. **373**, 132751 (2022). 10.1016/j.snb.2022.132751

[145] L. Song, W. Gao, Q. Han, Y. Huang, L. Cui et al., Construction of an aggregation-induced electrochemiluminescent sensor based on an aminal-linked covalent organic framework for sensitive detection of glutathione in human serum. Chem. Commun. **58**(75), 10524–10527 (2022). 10.1039/d2cc03753j 10.1039/d2cc03753j 36043554

[146] X.L. Mao, Q.X. Luo, Y.J. Cai, X. Liu, Q.Q. Jiang et al., Structural isomerism of covalent organic frameworks causing different electrochemiluminescence effects and its application for the detection of arsenic. Anal. Chem. **95**(28), 10803–10811 (2023). 10.1021/acs.analchem.3c02082 37401846 10.1021/acs.analchem.3c02082

[147] Q.Q. Jiang, Y.J. Li, Q. Wu, X. Wang, Q.X. Luo et al., Guest molecular assembly strategy in covalent organic frameworks for electrochemiluminescence sensing of uranyl. Anal. Chem. **95**(22), 8696–8705 (2023). 10.1021/acs.analchem.3c01299 37224420 10.1021/acs.analchem.3c01299

[148] Q.X. Luo, Y.J. Cai, X.L. Mao, Y.J. Li, C.R. Zhang et al., Tuned-potential covalent organic framework electrochemiluminescence platform for lutetium analysis. J. Electroanal. Chem. **923**, 116831 (2022). 10.1016/j.jelechem.2022.116831

[149] X. Ma, C. Pang, S. Li, Y. Xiong, J. Li et al., Synthesis of Zr-coordinated amide porphyrin-based two-dimensional covalent organic framework at liquid–liquid interface for electrochemical sensing of tetracycline. Biosens. Bioelectron. **146**, 111734 (2019). 10.1016/j.bios.2019.111734 31586759 10.1016/j.bios.2019.111734

[150] L. Li, W. Zhao, Y. Wang, X. Liu, P. Jiang et al., Gold nanocluster-confined covalent organic frameworks as bifunctional probes for electrochemiluminescence and colorimetric dual-response sensing of Pb^2+^. J. Hazard. Mater. **457**, 131558 (2023). 10.1016/j.jhazmat.2023.131558 37269568 10.1016/j.jhazmat.2023.131558

[151] R. Ma, J. Jiang, Y. Ya, Y. Lin, Y. Zhou et al., A carbon dot-based nanoscale covalent organic framework as a new emitter combined with a CRISPR/Cas12a-mediated electrochemiluminescence biosensor for ultrasensitive detection of bisphenol a. Analyst **148**(6), 1362–1370 (2023). 10.1039/d3an00024a 36857724 10.1039/d3an00024a

[152] L. Shen, Y.W. Wang, H.Y. Shan, J. Chen, A.J. Wang et al., Covalent organic framework linked with amination luminol derivative as enhanced ECL luminophore for ultrasensitive analysis of cytochrome C. Anal. Methods **14**(46), 4767–4774 (2022). 10.1039/d2ay01208a 36416105 10.1039/d2ay01208a

